# Synergistic Bactericidal Effects of R- and F-Type Pyocin Cocktails Against Clinical *Pseudomonas aeruginosa* Isolates from Central Taiwan

**DOI:** 10.3390/antibiotics15060596

**Published:** 2026-06-10

**Authors:** Yi-Luen Shen, Wen-Tong Xu, Zih-Ling Jiang, Nien-Jen Hu, Ying-Tsong Chen, Tze-Kiong Er, Chien-Wen Huang

**Affiliations:** 1 Division of Chest Medicine, Department of Internal Medicine, Asia University Hospital, Asia University, Taichung 413505, Taiwan; dioryrocky@gmail.com; 2 Doctoral Program in Translational Medicine, National Chung Hsing University, Taichung 402202, Taiwan; 3 Graduate Institute of Biochemistry, College of Life Sciences, National Chung Hsing University, Taichung 402202, Taiwan; a0983297879@gmail.com (W.-T.X.); njhu@nchu.edu.tw (N.-J.H.); 4 Taichung Chest Disease Care Association, Taichung 407219, Taiwan; cathleen8998@gmail.com; 5 Graduate Institute of Genomics and Bioinformatics, National Chung Hsing University, Taichung 402202, Taiwan; onion@nchu.edu.tw; 6 Institute of Molecular and Genomic Medicine, National Health Research Institutes, Miaoli 35053, Taiwan; 7 Division of Laboratory Medicine, Asia University Hospital, Asia University, Taichung 413505, Taiwan; 050016@tool.caaumed.org.tw; 8 Department of Medical Laboratory Science and Biotechnology, Asia University, Taichung 413505, Taiwan

**Keywords:** multiple drug resistant, *Pseudomonas aeruginosa*, pyocins, O-serotype distribution, lipopolysaccharide structure

## Abstract

**Background/Objectives**: *Pseudomonas aeruginosa* is a major cause of healthcare-associated infections, and the global rise of multidrug-resistant (MDR) strains has created an urgent need for alternative therapeutics. R- and F-type pyocins are phage tail-like bacteriocins that selectively kill *P. aeruginosa* by binding to lipopolysaccharide (LPS) receptors. We characterized O-serotype distribution and pyocin susceptibility among clinical isolates from central Taiwan to evaluate their therapeutic potential. **Methods**: A total of 109 ICU-derived *P. aeruginosa* isolates were analyzed. O-serotypes were determined by PCR, and pyocin gene carriage was confirmed by sequencing. Purified R1, R2, R5, F1, F2, F4, F7, and F12 pyocins were tested using spot assays. LPS profiles were examined by SDS-PAGE to explore structural correlates of resistance. Synergistic effects of combined R- and F-type pyocins were assessed in MDR isolates. **Results**: The most prevalent serotypes were O6 (23.9%), O2/O5/O16/O18/O20 (20.2%), O1 (16.5%), and O11/O17 (15.6%). Susceptibility was strongly serotype-dependent: O1 and O6 were highly sensitive to both pyocin types, whereas the O2/O5/O16/O18/O20 group showed marked resistance. SDS-PAGE demonstrated that resistant isolates possessed densely packed long-chain O-antigens, likely shielding LPS core receptors from pyocin binding. F-type pyocins exhibited bactericidal activity comparable to R-types, and R/F pyocin cocktails produced synergistic killing against MDR isolates. **Conclusions**: These findings provide an updated serotype profile of *P. aeruginosa* in Taiwan and highlight the importance of LPS structural variability in pyocin susceptibility. These results underscore the potential of pyocin-based cocktails as a promising precision-medicine strategy to inhibit the planktonic growth and biofilm formation of multidrug-resistant *P. aeruginosa* isolates.

## 1. Introduction

As a common nosocomial pathogen, the Gram-negative bacterium *Pseudomonas aeruginosa* frequently targets vulnerable populations, including those with immunodeficiency and cystic fibrosis [1]. Previous studies in Taiwan have identified *P. aeruginosa* as a major causative pathogen of healthcare-associated pneumonia, especially in severe cases requiring hospitalization or intensive care [2]. The rise and spread of multidrug-resistant (MDR) *P. aeruginosa* underscore the urgent need for novel therapeutic strategies that go beyond traditional antibiotics [3,4]. Beyond those novel therapeutics, pyocins present a highly specific and potent alternative for the precision treatment of bacterial infections, particularly against MDR *P. aeruginosa* strains [5,6,7].

Pyocins, a family of bacteriocins produced by *P. aeruginosa*, effectively kill bacterial strains of the same or closely related species [8]. Pyocins are generally classified into R-, F-, and S-type pyocins. R- and F-type pyocins are both phage tail-like bacteriocins (also called tailocins) [9]. They are structurally analogous to bacteriophages but have discarded the protective capsid and the encapsulated genetic materials. Both R- and F-type pyocins contain the receptor binding proteins (RBPs), also referred to as tail fibers, which specifically recognize the lipopolysaccharide (LPS) of target competitor bacteria [10,11,12]. In contrast, S-type pyocins are comparably smaller and water-soluble [8], consisting of a single polypeptide chain and function similarly to colicins, one type of narrow-spectrum bacteriocins produced by Enterobacteriaceae. S-type pyocins exert their bactericidal activities through DNase [13,14], tRNase [15], or pore-formation [16], and the molecular mechanisms of killing and immunity are relatively well-understood due to extensive studies on colicins.

R- and F-type pyocins can be further subtyped based on the amino acid sequence of their RBPs, with R-type pyocins classified into subtypes R1 to R5 [11], and F-type pyocins into subtypes F1 to F12 [12]. The killing mechanism of R-type pyocin action is better characterized compared to F-type pyocins. R-type pyocins are contractile bacterial nanomachines that share structural homology with the tails of Myoviridae bacteriophages. The headless particles kill target bacteria through a highly coordinated mechanism: tail fibers first recognize specific carbohydrate residues in the LPS core, triggering a sheath contraction that drives a central spike into the target cell. The physical puncture destroys the bacterial membrane potential and causes immediate cell death [10,17]. The killing mechanism of F-type pyocins remains under investigation, yet genetic and structural analyses suggest that they function similarly to Siphoviridae phage tails. Unlike their contractile R-type counterparts, F-type pyocins utilize a non-contractile mechanism to penetrate the bacterial membrane and dissipate the proton motive force [12].

LPS, a critical component of the outer membrane of Gram-negative bacteria, consists of three domains: lipid A, core oligosaccharides, and O-antigen polysaccharides [18]. While the lipid A and core regions are relatively conserved, the O-antigen is highly variable. *P. aeruginosa* synthesizes two types of O-antigens: the common polysaccharide antigen (CPA), composed of repeating units of D-rhamnose and conserved across most *P. aeruginosa* strains, and the O-specific antigen (OSA), which exhibits strain-specific variability in sugar composition [19,20]. The International Antigen Typing Scheme (IATS), the most widely used serotyping system for *P. aeruginosa*, classifies the bacteria into 20 serotypes (O1–O20) based on the structural diversity of OSA [18]. This classification plays a crucial role in epidemiological studies, clinical diagnostics, and prevalence tracking of specific *P. aeruginosa* strains. Previous studies have identified the LPS as the critical determinant for R-type pyocin activity, acting simultaneously as a protective shield and a target receptor [10,21]. However, there is a significant knowledge gap regarding whether F-type pyocins exhibit similar serotype-dependent specificity.

In this study, 109 clinical isolates of *P. aeruginosa* were collected from regional hospitals in Taichung, Taiwan. We employed PCR and DNA sequencing to identify their respective serotypes and characterized the specific pyocin profiles produced by each isolate using established LPS gene cluster data as a reference. These findings provide an updated epidemiological snapshot of the Taichung region in Taiwan and offer insights into the diversity of pyocin production among clinical strains. Furthermore, we purified three R-type and five F-type pyocins from the isolates and cross-mapped the susceptibility of the 109 isolates to these specific pyocins. The systematic study provides a comprehensive analysis of the correlation between the serotypes of clinical *P. aeruginosa* strains and susceptibility to R- and F-type pyocins. We also investigated the synergy between the R- and F-type pyocins against the planktonic growth and biofilm formation of MDR *P. aeruginosa*, highlighting the potential of phage tail-like pyocin-based cocktails to serve as precision antimicrobial agents with targeted killing activity against high-risk clinical strains.

## 2. Results

### 2.1. O-Serotype Distribution of the Clinically Isolated P. aeruginosa Strains in Central Taiwan

**Figure 1:**
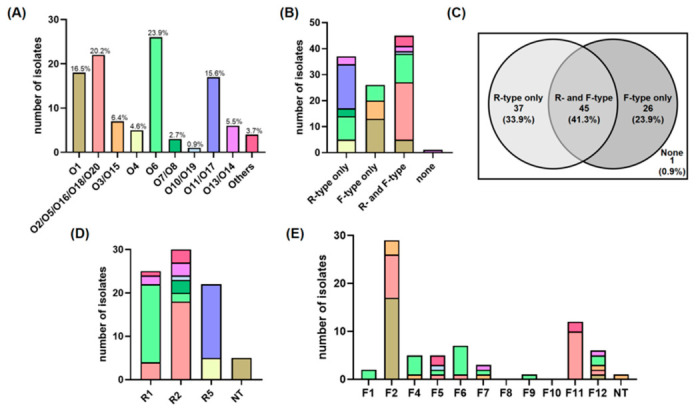
Distribution of O-antigen serotypes and pyocin subtypes among the clinical *P. aeruginosa* isolates. (**A**) Distribution of the 11 identified O-serotype groups, showing the absolute counts with prevalence rate determined via PCR-based serotyping. Pyocin production profiles across the isolate collection are presented by (**B**) a stacked histogram, and (**C**) a Venn diagram. The total number of isolates producing various (**D**) R-type and (**E**) F-type subtypes are shown. Each color in the stacked histogram corresponds to a specific O-antigen serotype as defined in (**A**). NT, non-typable.

A total of 109 clinical *P. aeruginosa* isolates were collected from the sterile sites of patients at two regional hospitals in Taichung, Taiwan. These isolates were serotyped using PCR with the primers as previously described by Raymond et al. [22] (Figure S1) due to the lack of a commercial antibody-based serotyping kit in Taiwan (see Table S1 for the sequences of primer pairs). This study demonstrated that the twenty *P. aeruginosa* serotypes (O1–O20) defined by the International Antigenic Typing System (IATS) can be grouped into 11 highly divergent gene clusters responsible for O-antigen biosynthesis of the LPS. However, the primer pair for determining O12 serotype was inadvertently omitted in the study of Raymond et al. [22]. To complement this, we used the primer pair reported by Thrane et al. to identify the serotype of O12 [23]. Among the 109 isolates, nine out of the 11 O-serotype groups were detected (Figure 1A), with the O9 and O12 groups being notably absent from the clinical isolates. Four isolates could not be typed using PCR-based serotyping and were classified as “Others”.

The statistical results revealed that the O6 serotype was the most prevalent among the 109 isolates, accounting for 23.9% (*n* = 26) of all the isolates (Figure 1A). This was followed by the O2/O5/O16/O18/O20 group (20.2%), O1 (16.5%), and the O11/O17 group (15.6%); the four dominant groups of serotypes represented 76.0% of the total isolates. The findings are largely consistent with prior studies [24,25] that identified O1, O2, O6, and O11 as the most frequent clinical serotypes, albeit with varying relative frequencies.

### 2.2. Profiling of R- and F-Pyocins in the P. aeruginosa Isolates

We performed molecular typing on the 109 isolates to determine the production of R- and F-type pyocins. The presence of the pyocin clusters was confirmed by PCR targeting the genes *PA0623* and *PA0633*, which encode the tube proteins of R- or F-type pyocins, respectively. The specific subtypes were then determined by PCR and DNA sequencing of the tail fiber genes *PA0620* (R-type) and *PA0641* (F-type) [10,12]. The amino acid sequences of the tail fibers for R2, R3, and R4 pyocins are extremely similar and are therefore referred to herein as R2 pyocin [26,27,28]. Among the 109 isolates, 33.9% (*n* = 37) carried genes for R-type pyocins exclusively, while 23.9% (*n* = 26) were restricted to F-type pyocins (Figure 1C). A substantial portion of the isolates (41.3%, *n* = 45) possessed both R- and F-type pyocin clusters. Notably, only one isolate (AUHPA48) lacked the genes producing any pyocin type and was labeled as “None” in Figure 1B. Among the 82 isolates carrying R-type pyocin gene clusters (Figure 1B), R2 pyocin was the most dominant subtype, found in 27.8% (*n* = 30) of the total collection (Figure 1D). This was followed by R1 and R5 pyocin subtypes, which were detected in 22.9% (*n* = 25) and 20.2% (*n* = 21) of the total isolates, respectively. These data indicate a relatively balanced distribution between the isolates carrying the various R-type pyocin subtypes and those lacking R-type pyocin genes (23.8%, *n* = 27). Consistent with the observations of Köhler et al. [10] and Mei et al. [5], our data revealed a high degree of specificity between pyocin subtypes and producer serotypes: 72.0% (18/25) of R1-producing isolates were serotype O6, 56.7% (17/30) of R2-producing isolates belonged to the serotype group O2/O5/O16/O18/O20, and 77.2% (17/22) of R5-producing isolates were O11/O17. Notably, five isolates (4.6%, *n* = 5) could not be typed using the primer pairs listed in Table S2, and these were classified as non-typable (NT) (Figure 1D). Interestingly, all five of these NT isolates belonged to the O1 serotype.

F-type pyocins comprise 11 subtypes (F1, F2, F4–F12), as described in a recent taxonomic study [12]. In contrast to the serotype distribution of R-pyocin producers, the prevalence of isolates carrying the F-pyocins exhibited a higher level of bias toward a specific pyocin subtype. Of the 71 isolates harboring F-type pyocin gene clusters (Figure 1B,C), the F2 subtype was predominant, accounting for 26.6% (*n* = 29) of the total collection, where 58.6% (17/29) of F2-producing isolates were serotype O1 (Figure 1E). This high prevalence is in line with previous reports identifying F2 as the most frequently occurring F-pyocin subtype [12]. This was followed by F11, which was identified in 11.0% (*n* = 12) of isolates. Other F-pyocins occurred at low frequencies, with each representing less than 10% of the isolates, or were entirely absent. Notably, one isolate (0.9%) failed to yield a product with the known F-type subtyping primers and was therefore classified as non-typable (NT) (Figure 1E).

### 2.3. The Correlation Between R- and F-Type Pyocin Susceptibility and O-Serotypes

To investigate the correlation between pyocin susceptibility and *P. aeruginosa* O-antigen profile, we conducted systematic spot assays to evaluate the R- and F-type pyocins’ susceptibility of 109 clinical isolates. A panel of eight pyocins (R1, R2, R5, F1, F2, F4, F7, and F12) was purified from the clinical isolates, AUHPA312, AUHPA318, AUHPA325, AUHPA331, AUHPA343, AUHPA348, AUHPA32, and AUHPA326, respectively, each of which exclusively produces a single pyocin subtype (Table S3).

**Figure 2:**
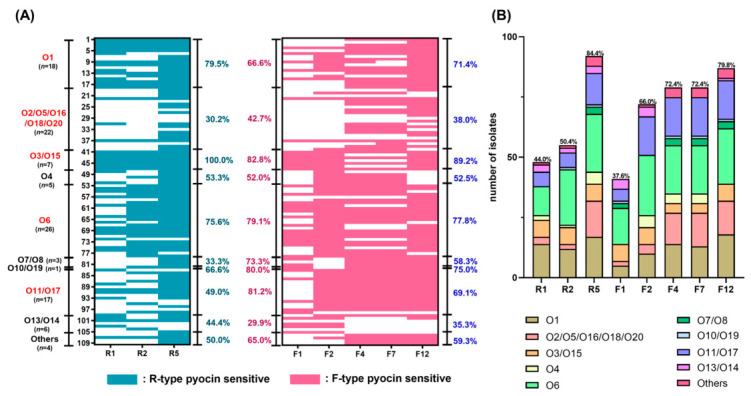
Bactericidal host range of pyocin subtypes against *P. aeruginosa* O-antigen serotypes. (**A**) Susceptibility profiles of 109 isolates to R-type (teal) and F-type (pink) pyocins, categorized by the eleven O-antigen serotype groups. The four predominant serotype groups are labeled red. The specific susceptibility rates to R- and F-pyocins are indicated in teal and pink, respectively, while combined susceptibility rates to both R- and F-type pyocins are highlighted in blue. (**B**) Prevalence of pyocin sensitivity across the collection. Histograms represent the total number of isolates, categorized as various serotypes, that are susceptible to each specific R- and F-type pyocin subtype. The color code is consistent with the scheme as defined in Figure 1.

Among the four serotype groups with higher prevalence in this study (O1, O2/O5/O16/O18/O20, O6, and O11/O17), O2/O5/O16/O18/O20 revealed the lowest susceptibility to the eight pyocins (30.2% and 42.7% to R- and F-pyocins, respectively) (Figure 2A, Table 1). The other three serotype groups (O1, O6, and O11/O17) exhibited high overall pyocin susceptibility rates with 71.4%, 77.8% and 69.1%, respectively. Particularly, a divergence in susceptibility was observed within the O11/O17 serotype group, with relatively low sensitivity to R-type pyocins (49.0%) but higher sensitivity to F-type pyocins (81.2%) (Figure 2A, Table 1). The results align with previous findings indicating that serotypes O1 and O6 are susceptible to R1, R2, and R5 pyocins, whereas serotype O11 displays high resistance [10]. Notably, the killing capacities of F-pyocins against isolates within the four major serotype groups were found to be comparable to or even greater than those of R-pyocins (Figure 2A, Table 1), highlighting that F-pyocins represent an antimicrobial agent as potent as R-pyocins [12]. The susceptibility screening revealed that R5 pyocin possessed the highest bactericidal efficacy, with 84.4% (*n* = 92) of the isolates being susceptible. This was followed by F12 pyocin, which exhibited 79.8% (*n* = 87) of the isolates (Figure 2B).

### 2.4. LPS Structural Variations Drive Differential R-Type Pyocin Susceptibility

Given that LPS can also function as a protective shield against R-type pyocins [10], we sought to determine whether structural variations in LPS contribute to the reduced susceptibility observed in the O2/O5/O16/O18/O20 serotype group. To investigate this, we performed SDS-PAGE analysis to characterize the LPS profiles of representative strains. Our analysis included five clinical isolates from the O6 serotype and five isolates from the O2/O5/O16/O18/O20 serotype group for comparative morphological characterization of LPS.

**Figure 3:**
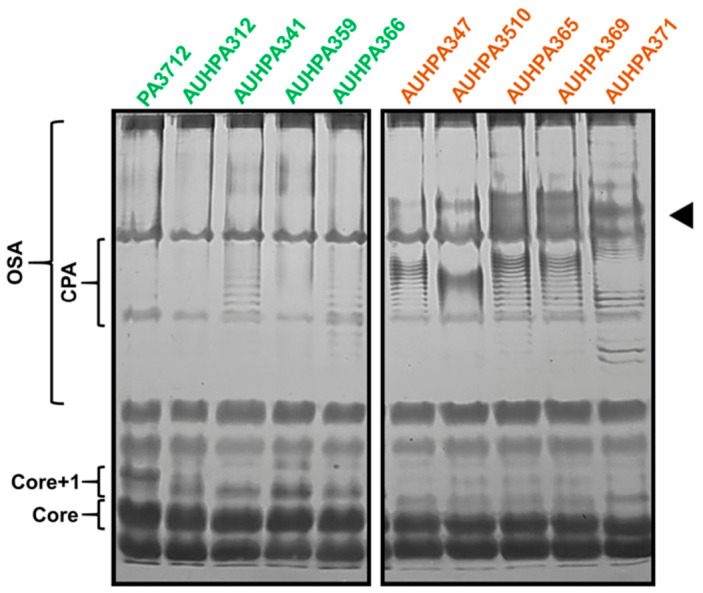
Comparative analysis of LPS profiles. Silver-stained SDS-PAGE of LPS extracted from clinical isolates belonging to the O2/O5/O16/O18/O20 serotype group (labeled in brown) and O6 serotype (labeled in green). The black triangle highlights the enrichment of high-molecular-weight OSA in the O2/O5/O16/O18/O20 serotype group. Brackets delineate the migration ranges of CPA and OSA. ‘Core’ denotes uncapped LPS, while ‘Core+1’ indicates the LPS core substituted with a single O-antigen repeating unit.

Electrophoretic analysis revealed that the majority of isolates exhibited a characteristic ladder-like pattern in the upper region of the gel, representing B-band (OSA) chains with varying O-antigen repeat numbers (Figure 3). Bands corresponding to the LPS core were identified at the migration front, alongside ‘core+1’ species—representing the core substituted with a single O-repeating unit. Notably, isolates within the O2/O5/O16/O18/O20 serotype group displayed significantly denser LPS packing and an enrichment of high-molecular-weight O-antigen chains compared to the O6 group (Figure 3, black triangle). This structural profile correlates with the observed resistance of these serotypes to the eight tested pyocins (Figure 1A, Table 1). Interestingly, the O2/O5/O16/O18/O20 group exhibited reduced levels of core+1 LPS relative to the O6 serotype. This shift from core+1 toward long-chain oligomerization in the resistant group highlights a structural basis for reduced pyocin susceptibility.

### 2.5. Synergistic Efficacy of R- and F-Pyocin Cocktails Against MDR P. aeruginosa Clinical Isolates

In light of the increasing prevalence of antibiotic-resistant *P. aeruginosa*, we evaluated the therapeutic potential of R- and F-type pyocins specifically against clinical MDR isolates. Among the total 109 isolates, 50 underwent antibiotic susceptibility testing, with the highest resistance rate observed for ciprofloxacin/levofloxacin (16.0%/20.0%), followed by imipenem/meropenem (10.0%/12.0%) (Table 2 and Table S4), consistent with the global survey of antibiotic resistance [24,29]. The screening led to the identification of three MDR isolates: AUHPA312, AUHPA325, and AUHPA399 (Table 2 and Table S4). Notably, all three MDR isolates exhibited susceptibility to at least one R- or F-type pyocin (Table S3), highlighting the potential of these bacteriocins as targeted antimicrobial agents for resistant infections.

**Figure 4:**
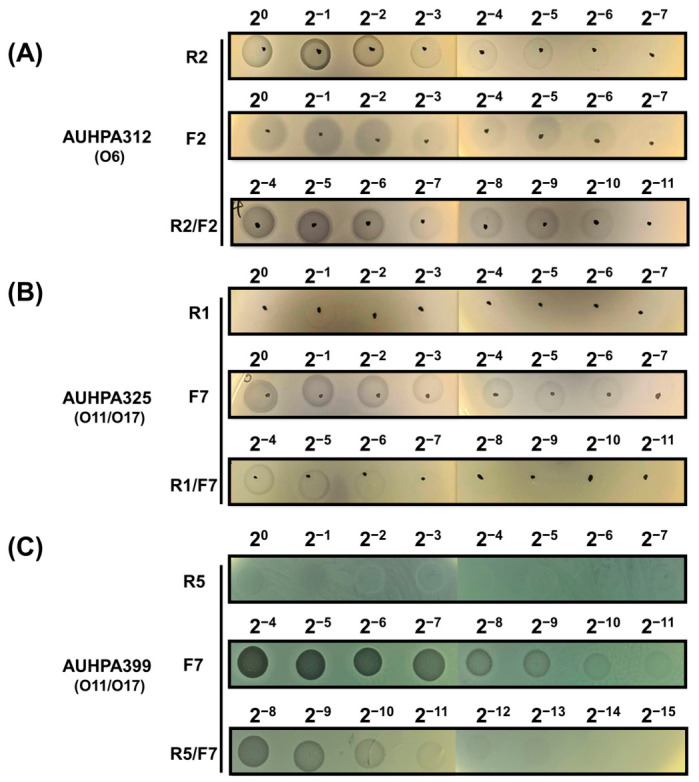
Synergistic bactericidal activity of pyocin cocktails against MDR *P. aeruginosa* isolate. Comparative bactericidal activities of individual R- and F-type pyocins versus their combined cocktail pairs against (**A**) AUHPA312, (**B**) AUHPA325, and (**C**) AUHPA399. Assays were performed using individual pyocins or cocktail pairs followed by twofold serial dilution as indicated.

To assess the bactericidal efficacy of pyocin cocktail therapeutics against MDR *P. aeruginosa*, we evaluated combinations of R- and F-type pyocins against isolates AUHPA312 and AUHPA325. To maximize the likelihood of a potent effect, we restricted our cocktail components to individual pyocins that were independently active against both target strains. By systematically paring these R- and F-type variants (Figure 4), we were able to evaluate their interactions. The nature of these interactions—whether synergistic, additive, indifferent, or antagonistic—was characterized using fractional inhibitory concentration index (FICI) analysis (Table 3 and Table S5).

Isolate AUHPA312 was identified as O6 serotype and carried the R1 pyocin gene cluster. As expected, this isolate exhibited resistance to R1 pyocin (Table S3). This is consistent with the principle that producer strains are typically immune to their own pyocin type [8,10,11,12], although the underlying mechanism governing this self-immunity remains to be elucidated. Consequently, R1 pyocin was excluded from the cocktail formulations (Table 3). Specifically, the parings of R2/F1, R2/F2, or R2/F12, R5/F1, and R5/F2, demonstrated potent synergy, as evidenced by the FICI values ≤ 0.5 (Figure 4, Table 3). For isolate AUHPA325 (O11/O17 serotype, R5 pyocin carrier), R5 pyocin was excluded from synergy assays due to self-resistance (Table S3). Additionally, R2 was also omitted from testing because the strain was inherently resistant to R2 (Table S3). However, all of the tested cocktail formulations against AUHPA325 resulted only in additive interactions, with no synergistic effects detected (Figure 4, Table 3). Interestingly, although isolate AUHPA399 (O11/O17 serotype) is an R5 pyocin carrier, it showed susceptibility to R5 pyocin, which is inconsistent with the self-immunity dogma; however, this kind of self-sensitivity was observed by Mei et al., probably due to the mutations of the LPS receptor [5]. AUHPA399 was resistant to R2 and F1 (Table S3), and therefore, the two pyocins were excluded from synergy assays. The pairing of R5/F7 demonstrated a synergistic killing effect with FICI = 0.3002 (Figure 4, Table 3).

**Figure 5:**
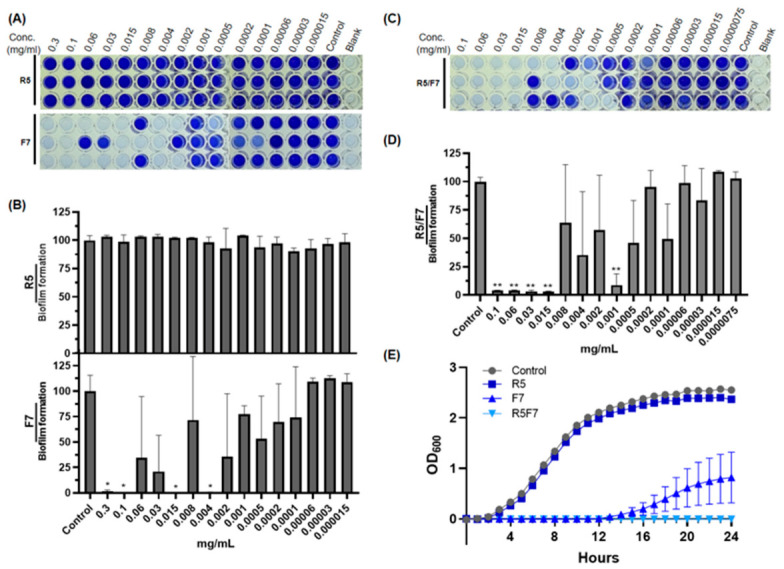
Biofilm development and planktonic growth of AUHPA399 after pyocin treatments. (**A**,**C**) Crystal violet assays showing biofilm formation of AUHPA399 after treatments of (**A**) individual pyocins, R5 and F7, and (**C**) R5/F7 cocktail. The highest concentrations of added pyocin are indicated, followed by 2-fold serial dilution. Control: no pyocin added. (**B**,**D**) Normalized level of biofilm formation of AUHPA399 in 2-fold serially diluted (**B**) individual pyocins R5 and F7, and (**D**) R5/F7 cocktail. (**E**) Bacterial growth kinetics of AUHPA399 after pyocin treatments. All the OD_570_ values in each well are normalized against the control as 100%. The statistical significance between the OD_570_ values for the control and pyocin-treated cells was analyzed by one-way ANOVA using GraphPad Prism 9. Data represent the mean ± SD with *n* = 3. * *p* < 0.05, ** *p* < 0.01.

To evaluate the capability of the pyocins to inhibit biofilm formation, crystal violet assays were performed on the two multidrug-resistant (MDR) isolates, AUHPA312 and AUHPA399, following treatment with the R2/F2 and R5/F7 cocktails, respectively (Figure 5 and Figure S2). Unexpectedly, AUHPA312 exhibited no pyocin concentration-dependent reduction in biofilm formation when treated with individual R2, F2, or the combined R2/F2 cocktail (Figure S2). Conversely, AUHPA399 showed complete inhibition and a dose-dependent decrease in biofilm mass when treated with individual F7 or the R5/F7 cocktail (Figure 5A–D). Although some heterogeneity in biofilm development was noted across the three biological replicates—potentially due to pyocin self-aggregation or receptor competition—the MICs required to completely suppress biofilm development were determined to be 0.004 mg/mL for the F7 pyocin and 0.001 mg/mL for the R5/F7 cocktail (Figure 5B,D). Furthermore, growth kinetics experiments demonstrated that while R5 pyocin exhibited very mild bactericidal activity, F7 pyocin exerted a potent effect, and the combined R5/F7 cocktail completely suppressed bacterial growth throughout the 24-h observation period (Figure 5E).

## 3. Discussion

The O-antigen in *P. aeruginosa* LPS is a critical virulence factor that facilitates systemic dissemination [30,31,32]. Accordingly, characterizing serotype distribution is vital for optimizing clinical management and developing serotype-specific therapeutic interventions. Given that the most recent epidemiological data from Taiwan dates back to 1988 [33], predating the widespread adoption of the IATS classification system, in this study, we updated the regional epidemiological profile by serotyping 109 isolates from central Taiwan. Our serotyping analysis indicated that O6, O1, O2/O5/O16/O18/O20, and O11/O17 are the most predominant serotypes (Figure 1). These results largely align with the global epidemiological data reported by Pirnay et al. [34] and Nasrin et al. [29], which identified O11, O1, and O6 as the top three most prevalent serotypes via traditional antibody-based slide agglutination assays. Notably, when Nasrin et al. employed PCR to characterize isolates that were non-typable by agglutination assay [35], the O2/O5/O16/O18/O20 group emerged as the second most frequent. This underscores the value of PCR-based serotyping as a critical tool to complement traditional serotyping assays. Serotypes O2, O5, O16, O18, and O20 exhibit significant structural similarities in their O-antigen architecture [20,35,36]. As demonstrated by Raymond et al. [22], these five serotypes constitute a group due to a high degree of DNA genetic similarity. While current PCR-based serotyping lacks the resolution to differentiate individual members of this group, it remains a more accessible alternative for general laboratories. However, further investigation into the fine-scale genetic variations of the LPS biosynthesis gene cluster is required to refine PCR- or sequencing-platforms for higher-resolution serotyping.

We identified the O2/O5/O16/O18/O20 serogroup as the least susceptible to pyocin-mediated killing. This resistance is consistent with the model where long-chain O-antigens in strains like PAO1 (serotype O5) shield the LPS core receptors [10], a phenomenon previously visualized via electron microscopy and silver-stained SDS-PAGE as high-density LPS packing [37]. For the first time, we provide a systematic comparison of F- and R-pyocin spectra, demonstrating that both types exhibit a shared sensitivity profile (O1, O6, and O11) and resistance profile (O2/O5/O16/O18/O20). While these susceptibility patterns align with the findings on F-pyocins reported by Saha et al. [12], the precise molecular receptors for F-pyocins have yet to be determined.

The strategies employed by R- and F-type pyocins to prevent self-killing differ fundamentally from those of S-type pyocins [8]. S-type pyocins function analogously to colicins, killing target cells by degrading DNA (pyocins AP41, S1, S2, and S3), hydrolyzing tRNA (pyocin S4), or permeabilizing the cytoplasmic membrane (pyocin S5). To ensure self-protection, a short immunity gene is co-expressed alongside the cognate S-type pyocin; this immunity protein binds directly to the cytotoxic domain and neutralizes its bactericidal activity. Conversely, R- and F-type pyocins lack dedicated immunity proteins, and the precise mechanisms by which producing strains evade their own tail-like bacteriocins remain a subject of debate. Systematic mutational analyses involving the deletion of the O-specific antigen (OSA), common antigen (CPA), or core oligosaccharide have demonstrated that OSA and CPA polysaccharide chains sterically shield the cell from R-type pyocins, whereas the outer core oligosaccharide serves as the primary receptor [10]. In contrast, the specific receptor configuration required for F-type pyocin recognition remains less conclusive [12].

Standard empirical therapies, though critical for improving survival rates in severe *P. aeruginosa* infections, are increasingly undermined by the bacterium’s extensive resistance profile. This clinical crisis has led the World Health Organization to list carbapenem-resistant *P. aeruginosa* (CRPA) among the three species requiring the most urgent antibiotic development [38]. Recent surveillance data from Taiwan indicates a significant upward trend in the prevalence of CRPA (10.2%, 349 out of 3408 isolates) [39]. A primary molecular driver for this resistance is the carriage of carbapenemase and efflux pump-encoding genes [3]. Phage tail-like pyocins can bypass the microbial defense mechanisms by leveraging O-antigen specificity for precision targeting [40].

To the best of our knowledge, this work provides the first documented evidence of the bactericidal efficacy of combined R- and F-type pyocin formulations. In the present study, the three MDR isolates, AUHPA312 (serotype O6), AUHPA325 (serotype O11/O17), and AUHPA399 (serotype O11/O17), displayed susceptibility to at least one R- and F-pyocin, underscoring their therapeutic potential against *P. aeruginosa* infections. Furthermore, R- and F-pyocin cocktails yielded synergistic bactericidal activity against AUHPA312 but remained additive against AUHPA325. This discrepancy may be attributed to the susceptibility profile of AUHPA325, which showed resistance to both R2 and R5 pyocins, and weak susceptibility to R1 pyocin. Given that this isolate was strongly susceptible to F-pyocins (Table S3), the dominant killing activity of F-pyocins likely obscured the detection of synergy between the two pyocin classes. The high susceptibility of the O11/O17 group, including isolate AUHPA325, to F-pyocins (Figure 2A, Table 1) correlates with their genomic architecture. As illustrated in Figure 1B, these strains possess only R-type pyocin gene clusters. Because bacteria often evolve immunity primarily against the specific class of pyocins they produce themselves, the exclusive presence of R-pyocin genes in the O11/O17 group provides a plausible explanation for their vulnerability to exogenous F-pyocins (Figure 1B).

Although the R2/F2 pyocin cocktail produced discernible zones of inhibition on a lawn of AUHPA312 (Figure 4), substantial biofilm development was still observed in TSB medium (Supplementary Figure S2). Notably, the inhibition zones for AUHPA312 were less distinct than those for AUHPA399, suggesting incomplete bactericidal activity. We hypothesize that partially lysed cells enhance the release of extracellular DNA (eDNA), which then serves as a structural scaffold that facilitates the adherence and survival of remaining cells during biofilm development. A similar phenomenon has been reported under sub-lethal concentrations of conventional antibiotics [41]. To our knowledge, this is the first study to document such biofilm-promoting concerns regarding R- and F-type pyocins. Consequently, the risk of induced biofilm formation must be carefully evaluated alongside standard bactericidal assays when characterizing these tail-like bacteriocins for therapeutic development.

## 4. Materials and Methods

### 4.1. P. aeruginosa Strains

In this study, 101 clinical isolates of *P. aeruginosa* were obtained from Asia University Hospital, Taichung, Taiwan, and eight isolates were collected from Feng Yuan Hospital, Taichung, Taiwan [42,43]. A total of 109 isolates were independently collected from sterile sites. All isolates were preserved in Tryptic Soy Broth (TSB) containing 25% (*v*/*v*) glycerol and stored at −80 °C. According to the Clinical and Laboratory Standards Institute guidelines, isolates exhibiting resistance to antibiotics from three or more different classes with distinct mechanisms of action are defined as MDR.

### 4.2. O-Antigen Serotype Determination of P. aeruginosa

The O-antigen serotyping of *P. aeruginosa* was conducted using PCR followed by DNA sequencing as described by Raymond et al. [22] (Figure S1, Table S1). Genomic DNA from the isolates was extracted using the EasyPrep Genomic DNA Extraction Kit (Biotools, New Taipei City, Taiwan) following the manufacturer’s instructions. The primer sequences used for O-antigen serotyping are summarized in Table S1.

### 4.3. Identification of R- and F-Type Pyocin Subtypes

Identification and subtyping of R- and F-type pyocins were performed using PCR followed by sequencing where necessary. The primer pairs used for pyocin identification and subtyping are summarized in Table S2. The presence of R- and F-type pyocins in the isolates was confirmed by PCR amplification of the tube genes of R-type (*PA0623*) and F-type pyocins (*PA0633*). To determine the subtypes of R-type pyocin in the isolates, the tail fiber gene (*PA0620*) was specifically amplified using primer pairs targeting the R1, R2 group (comprising R2, R3, R4), and R5. For F-type pyocin subtyping, the DNA fragments from the gene encoding the central tail fiber (*PA0641*) to *trpG* (PA0649) were amplified, followed by Sanger sequencing. The sequenced data were mapped against the amino acid sequences of PyoF10 of various F-pyocins as reported by Saha et al. [12].

### 4.4. Purification of R- and F-Type Pyocin

Purification of R- and F-type pyocins was performed with modifications to the methods described by Ge et al. [44]. For pyocin susceptibility testing, strains AUHPA312, AUHPA318, AUHPA325, AUHPA331, AUHPA343, AUHPA348, AUHPA32, and AUHPA326 were used as the producers for R1, R2, R5, F1, F2, F4, F7, and F12 pyocins, respectively. A single colony of each pyocin-producing strain was inoculated into 20 mL of TSB and incubated in a shaker incubator overnight at 37 °C, 220 rpm. The culture was then transferred to 400 mL of fresh TSB and incubated for two hours at 37 °C. To induce the production of pyocins, mitomycin C (3 µg/mL) was then added to trigger the SOS response of *P. aeruginosa*, followed by incubation for another two and a half hours at 30 °C. DNase I was added to the final concentration of 3 μg/mL, with incubation for another 30 min. The culture was centrifuged, and the supernatant was filtered through a 0.22 µm filter. Ammonium sulfate was added to a final concentration of 50% (*w*/*v*) to precipitate the pyocins. After one hour at 4 °C, the precipitated protein was collected by centrifugation at 10,000× *g* for 15 min, resuspended in SM buffer (50 mM Tris-HCl, pH 7.5, 100 mM NaCl, and 8 mM MgCl_2_), and dialyzed against SM buffer overnight. The sample was then centrifuged at 150,000× *g* for 1 h at 4 °C. Pyocins were fractionized in the pellets and resuspended in 2 mL of SM buffer. The protein concentration was measured using the Pierce™ BCA Protein Assay Kit (Thermo Scientific, Waltham, MA, USA). The purified R- or F-type pyocins were stored at −80 °C.

### 4.5. Susceptibility Assay of P. aeruginosa to R- and F-Pyocin

A single colony of each test isolate was inoculated into TSB and incubated overnight at 37 °C, 220 rpm. An aliquot of 100 µL from the overnight culture was added to TSB soft agar (0.75% agar), thoroughly mixed, and poured into a Petri dish to solidify. Then, 10 µL of R- and F-type pyocins, each at a concentration of 0.5 mg/mL, was spotted onto the solidified soft agar surface. The plates were incubated overnight at 37 °C. To evaluate the efficacy of the pyocin cocktail combination against MDR strains, the bactericidal activity of individual pyocins was first assessed using twofold serial dilutions. Next, individual R- and F-type pyocins of different subtypes were mixed at a ratio of 1:1 (*v*/*v*), and the bactericidal activity of the combination was evaluated using the same method. The synergy of the cocktail combinations was assessed using the fractional inhibition concentration index (FICI). A clear zone of inhibition on the plate was considered indicative of sensitivity, while a faint zone indicated weak sensitivity. The formation of plaques was interpreted as phage contamination.

### 4.6. LPS Extraction and Visualization

The extraction and visualization of LPS were performed as described by Kulikov et al. [45] with minor modifications. In brief, one milliliter of overnight TSB culture of selected *P. aeruginosa* strains was pelleted by centrifugation at 10,000× *g* for 30 s using a benchtop microcentrifuge, washed three times with an identical volume of PBS and subsequently resuspended in 50 μL of lysis buffer (2% *w*/*v* of SDS, 4% *v*/*v* of 2-mercaptoethanol, 10% *v*/*v* glycerol, 1 M Tris-HCl (pH 6.8), and 0.01% bromophenol blue). After incubation at 95 °C for 10 min followed by cooling to room temperature, the lysate was treated with the final concentration of 2.5 mg/mL protease K in the lysate. The mixture was then incubated for 1 h in a thermomixer. Two microliters of the sample was loaded onto a 12% SDS-PAGE gel (29:1 acrylamide:bis-acrylamide) for electrophoresis. After electrophoresis, the gel was incubated for 15 min in fixer-oxidizer solution, followed by three washes with distilled water (7 min each). The gel was stained using the ProteoSilver™ Silver Stain Kit (Sigma, St. Louis, MO, USA).

### 4.7. Kinetic Assay

AUHPA399 was cultured overnight in TSB broth at 37 °C with shaking at 220 rpm. The overnight culture was diluted with fresh TSB and mixed with R5, F7, or the R5F7 combination in a 96-well microtiter plate, resulting in a final bacterial density of OD_600_ = 0.01 and a final pyocin concentration of 0.004 mg/mL. Bacterial growth was monitored using a microplate reader (BMG LABTECH SPECTROstar Nano, Ortenberg, Germany) at 37 °C with orbital shaking at 200 rpm for 24 h, and OD_600_ values were recorded every hour.

### 4.8. Microtiter Biofilm Inhibition Assay

The bacterial strain was cultured overnight (O/N) in TSB broth at 37 °C with shaking at 220 rpm. The overnight culture was diluted with fresh TSB and mixed with different concentrations of individual R-type and F-type pyocins or their cocktail in a 96-well microtiter plate, resulting in a final bacterial density of OD_600_ = 0.01. The plate was incubated statically at 37 °C for 24 h. After incubation, planktonic cells and culture medium were removed, and the wells were gently washed twice with phosphate-buffered saline (PBS) to remove non-adherent cells. The attached biofilms were stained with 0.1% crystal violet (CV) for 15 min, followed by two PBS washes to remove excess stain. The bound CV was then solubilized with 30% acetic acid for 15 min at room temperature, and the absorbance was measured at 570 nm (OD_570_) using a microplate reader to evaluate the inhibitory effects of individual R-type and F-type pyocins and their cocktail on biofilm formation.

## 5. Conclusions

In summary, this study provides an updated serotype distribution of *P. aeruginosa* serotypes in central Taiwan and identified F-type pyocins as potent antimicrobial candidates against clinical isolates, potentially surpassing R-type pyocins in efficacy. Our analysis characterized the distinct associations between serotype profiles and pyocin susceptibility, demonstrating that variations in LPS are primary drivers of the observed resistance patterns. While the precise mechanisms of receptor recognition and self-immunity require further investigation, these findings underscore the therapeutic potential of pyocin cocktails as a robust strategy for combating MDR *P. aeruginosa*.

## Figures and Tables

**Table 1:** Susceptibility of 109 *P. aeruginosa* isolates, categorized as O-antigen serotype, to various pyocin subtypes.

Group	Number of Isolates	R1	R2	R5	F1	F2	F4	F7	F12
O1	18	14 (77.7%)	12 (66.6%)	17 (94.4%)	5 (27.7%)	10 (55.5%)	14 (77.7%)	13 (72.2%)	18 (100%)
O2/O5/O16/O18/O20	22	3 (13.6%)	2 (9.1%)	15 (68.1%)	2 (9.1%)	4 (18.1%)	13 (59.1%)	14 (63.6%)	14 (63.6%)
O3/O15	7	7 (100%)	7 (100%)	7 (100%)	7 (100%)	7 (100%)	4 (57.1%)	4 (57.1%)	7 (100%)
O4	5	2 (40%)	1 (20%)	5 (100%)	0 (0%)	5 (100%)	4 (80%)	4 (80%)	0 (0%)
O6	26	12 (46.1%)	23 (88.4%)	24 (92.3%)	15 (57.6%)	25 (96.1%)	20 (76.9%)	20 (76.9%)	23 (88.4%)
O7/O8	3	0 (0%)	0 (0%)	3 (100%)	2 (66.6%)	0 (0%)	3 (100%)	3 (100%)	3 (100%)
O10/O19	1	0 (0%)	1 (100%)	1 (100%)	1 (100%)	0 (0%)	1 (100%)	1 (100%)	1 (100%)
O11/O17	17	6 (35.3%)	6 (35.3%)	13 (76.5%)	5 (29.4%)	16 (94.1%)	16 (94.1%)	16 (94.1%)	16 (94.1%)
O13/O14	6	3 (50%)	2 (33.3%)	3 (50%)	4 (66.6%)	4 (66.6%)	0 (0%)	0 (0%)	1 (16.6%)
Others	4	1 (25%)	1 (25%)	4 (100%)	0 (0%)	1 (25%)	4 (100%)	4 (100%)	4 (100%)

**Table 2:** Antibiotic resistance profiles of 50 *P. aeruginosa* isolates. The sensitivity (S) and resistance (R) status for the MDR isolates AUHPA312, AUHPA325, and AUHPA399 are indicated for comparison.

Antibiotics	Resistant/Total Strains	AUHPA312	AUHPA325	AUHPA399
Ciprofloxacin	8/50 (16.0%)	R	R	S
Levofloxacin	10/50 (20.0%)	R	R	R
Ceftazidime	3/50 (6.0%)	R	S	S
Cefepime	3/50 (6.0%)	R	R	S
Imipenem	5/50 (10.0%)	R	S	R
Meropenem	6/50 (12.0%)	R	R	R
Piperacillin/Tazobactam	3/50 (6.0%)	R	S	S

**Table 3:** A Synergy analysis of R- and F-pyocin cocktails against MDR *P. aeruginosa* isolates AUHPA312, AUHPA325, and AUHPA399 based on FICI.

AUHPA312 (O6)			AUHPA325 (O11/O17)			AUHPA399 (O11/O17)		
Cocktail Formula	FICI	Interpretation	Cocktail Formula	FICI	Interpretation	Cocktail Formula	FICI	Interpretation
R2/F1	0.2125	S	R1/F1	3.33	Ind	R1/F2	0.5003	Add
R2/F2	0.09375	S	R1/F2	0.5025	Add	R1/F4	0.50005	Add
R2/F4	0.50125	Add	R1/F4	0.52	Add	R1/F7	1.0006	Ind
R2/F7	0.50125	Add	R1/F7	0.52	Add	R1/F12	3.3336	Ind
R2/F12	0.283	S	R1/F12	0.50015	Add	R5/F2	5.003	Anta
R5/F1	0.225	S				R5/F4	6.6673	Anta
R5/F2	0.125	S				R5/F7	0.3002	S
R5/F4	0.5025	Add				R5/F12	6.6673	Anta
R5/F7	0.5025	Add						
R5/F12	0.533	Add						

FICI: fractional inhibitory concentration index. FICI ≤ 0.5, synergy, S; 0.5 < FICI ≤ 1, additive, Add; 1 < FICI ≤ 4, indifferent, Ind; FICI > 4, antagonism, Anta.

## Data Availability

The data supporting the findings of this study are available from the corresponding author, C.-W. Huang, upon reasonable request.

## Supplementary Materials

The following supporting information can be downloaded at: https://www.mdpi.com/article/10.3390/antibiotics15060596/s1, Figure S1: A schematic diagram of the 11 groups of gene clusters involved in OSA biosynthesis [46]; Figure S2: Biofilm development of AUHPA312 after pyocin treatments; Table S1: Primers used for serotyping; Table S2: Primers used for R- and F-type pyocin subtyping; Table S3: Serotype, R-, and F-type pyocin production and susceptibility of 109 isolates; Table S4: Antimicrobial susceptibility testing of 50 isolates; Table S5: Minimum inhibitory concentrations (MICs) of pyocin subtypes against AUHPA312 and AUHPA325.

## Abbreviations

The following abbreviations are used in this manuscript:

CPA	Common polysaccharide antigen
FICI	Fractional inhibition concentration index
OSA	O-specific antigen
LPS	Lipopolysaccharide
MDR	Multidrug resistant
