# Supplementary material for: Synergistic Bactericidal Effects of R- and F-Type Pyocin Cocktails Against Clinical Pseudomonas aeruginosa Isolates from Central Taiwan
Source: Antibiotics (Basel). 2026 Jun 10;15(6):596. doi: 10.3390/antibiotics15060596 (PMC13295577; doi:10.3390/antibiotics15060596)
Supplement: Supplementary file 1 [file antibiotics-15-00596-s001.zip › antibiotics-4270305-supplementary.pdf]

Table S1. Primers used for *P. aeruginosa* serotyping.

Primer name	Primer sequence (5'-3')	Product size
O1-F<br>O1-R	TGCTCTTTGGCTTCTTGATTCTTG<br>TCTACACCGCCAGAACTACCTAGCT	397
O2/O5/O16/O18 /O20-F<br>O2/O5/O16/O18 /O20-R	GCGGCTTTTATCAACCGTGTCGCA<br>TCCCTTGGCACACTGGAAGGACAT	1218
O3/O15-F<br>O3/O15-R	GCGTCGTTGTTCAGTTTGGACGTG<br>TCTGGAAAACCTGAGCAGCCGTCC	407
O4-F<br>O4-R	GCTAATAACGGAAGGACCTTGAAT<br>TAAAAGCTCGGCGTAACGCTTATG	552
O6-F<br>O6-R	ATTGGCTAGTGCTACACGAGTGCA<br>CGAATTAGCTTGCTCTTCAGGAAAG	381
O7/O8-F<br>O7/O8-R	GGAATGTCGCTCTCGTTTCAAGTG<br>CTAGTATTCATCAACTGCTGTAC	452
O9-F<br>O9-R	GGAACATTGGAATCAAGAGGTTATG<br>CCACTAAATACCAGGCATACAACTA	555
O10/O19-F<br>O10/O19-R	CAGCAGGGAATATCGCTTGAACAGT<br>CTATAATGCATTAGCGACTCACCG	548
O11/O17-F<br>O11/O17-R	TACTTCACCCATAGCTAGCGCTCTA<br>TTCTCTCTCAACTTAACCGTGGCC	453
O12-F<br>O12-R	ATGAAAAAAGTTTTGGTTACTGGG<br>CCCTCTCGAATCGAGTAGGTAGGCTC	905
O13/O14-F<br>O13/O14-R	CTATTCAGTCGATGATCCTTGTG<br>AGCAGTTAGCAAATTTCACTCTCCAGCC	489

Table S2. Primers used for R- and F-type pyocin subtyping.

Primer name	Primer sequence (5'-3')	Product size
R pyocin tube-F<br>R pyocin tube-R	ATGATTCCGCAAACCCTGACC<br>TTACAGGCCGAGGTCGTTGC	504
F pyocin tube-F<br>F pyocin tube-R	ATGTCCATCCTGACTCAAGGTACC<br>TCAAGCCGACTTCGGCGTC	495
F pyocin typing-F<br>trpG-R	CAGTTGATCACCCAGGAGTACGTC<br>GCACCAGGTTGTAGGTGAAGG	4k-5k
F pyocin sequencing	GCAGGTGGCGAGCCATAC	none
R pyocin typing-F	GATGTATGTACGGGTGTCCTACGC	
R 1-R	ATGCCATAGTTTTGAGCCAGTCCGTAG	831
R 2-R	CCGCCTGAGCAAAGTAGCC	797
R 5-R	CGCATAGTTCTGGACCGCACCGTG	832

Table S3. Serotypes, pyocin subtypes and susceptibility of 109 isolates.

Strains	Serotype	R-pyocin type	F-pyocin type	Sensitivity							
				R1	R2	R5	F1	F2	F4	F7	F12
AUHPA311	Others	R2	F11	R	R	(S)	R	R	S	S	(S)
AUHPA312	O6	R1	×	R	(S)	(S)	S	S	S	S	(S)
AUHPA313	O2/O5/O16/O18/O20	R2	F11	R	R	(S)	R	R	R	R	(S)
AUHPA314	O2/O5/O16/O18/O20	R2	F11	R	R	(S)	R	(S)	(S)	(S)	R
AUHPA315	O3/O15	×	F2	S	S	(S)	(S)	S	S	S	S
AUHPA318	O13/O14	R2	×	(S)	R	(S)	(S)	S	R	R	(S)
AUHPA319	O4	R5	×	(S)	R	(S)	R	(S)	R	R	R
AUHPA321	O1	×	F2	S	(S)	(S)	R	R	(S)	(S)	S
AUHPA322	O1	×	F2	S	(S)	(S)	R	R	R	R	S
AUHPA323	O1	×	F2	S	(S)	(S)	R	R	(S)	(S)	S
AUHPA325	O11/O17	R5	×	(S)	R	R	(S)	S	(S)	(S)	S
AUHPA326	O3/O15	×	F12	S	S	(S)	S	S	R	R	(S)
AUHPA331	O6	×	F1	S	S	(S)	R	S	S	S	S
AUHPA332	O2/O5/O16/O18/O20	R2	F2	R	R	(S)	R	R	R	R	R
AUHPA333	O13/O14	R1	×	(S)	S	(S)	R	R	R	R	R
AUHPA334	O1	NT	F2	S	(S)	S	(S)	S	S	S	(S)
AUHPA335	O2/O5/O16/O18/O20	R1	F6	(S)	S	(S)	(S)	(S)	S	S	(S)
AUHPA336	O6	R1	×	(S)	S	(S)	R	S	(S)	(S)	R
AUHPA337	O2/O5/O16/O18/O20	R2	F11	R	R	R	R	R	(S)	(S)	(S)
AUHPA339	O11/O17	R5	×	R	(S)	(S)	R	(S)	(S)	(S)	S
AUHPA3310	O7/O8	R2	×	R	R	(S)	S	R	S	S	S
AUHPA341	O6	R1	×	R	S	(S)	S	S	S	S	S
AUHPA342	O1	×	F2	S	(S)	(S)	R	R	(S)	(S)	S
AUHPA343	O1	×	F2	(S)	R	R	(S)	S	S	S	(S)
AUHPA344	O11/O17	R5	×	R	R	(S)	R	(S)	S	S	S

AUHPA345	O2/O5/O16/O18/O20	R2	F11	R	R	(S)	R	R	(S)	(S)	(S)
AUHPA346	O1	NT	F2	R	R	(S)	R	(S)	R	R	S
AUHPA347	O2/O5/O16/O18/O20	R2	F2	R	R	(S)	R	R	R	R	S
AUHPA348	O6	×	F4	(S)	(S)	(S)	R	R	R	R	(S)
AUHPA349	O11/O17	R5	×	R	R	(S)	S	(S)	S	S	S
AUHPA3410	O13/O14	R2	F12	R	R	R	(S)	S	R	R	R
AUHPA351	O6	R2	×	R	R	(S)	R	(S)	S	S	S
AUHPA352	O6	R1	F6	R	(S)	(S)	(S)	S	(S)	(S)	S
AUHPA354	O6	R1	F6	(S)	S	(S)	S	(S)	R	R	S
AUHPA355	O4	R5	×	R	R	(S)	R	(S)	(S)	(S)	R
AUHPA356	O11/O17	R5	×	R	(S)	(S)	R	S	S	S	S
AUHPA357	O1	×	F2	R	R	(S)	R	(S)	(S)	(S)	S
AUHPA359	O6	R1	×	R	(S)	(S)	S	S	(S)	(S)	S
AUHPA3510	O2/O5/O16/O18/O20	R2	F2	R	R	R	R	R	(S)	S	(S)
AUHPA361	O2/O5/O16/O18/O20	R2	F11	R	R	(S)	R	R	(S)	(S)	(S)
AUHPA362	O11/O17	R5	×	(S)	R	(S)	R	(S)	(S)	(S)	S
AUHPA363	O11/O17	R5	×	R	R	(S)	R	(S)	S	S	S
AUHPA365	O2/O5/O16/O18/O20	R2	F2	R	R	R	R	R	R	R	(S)
AUHPA366	O6	R1	F6	R	(S)	(S)	S	S	S	S	S
AUHPA367	O6	R1	F4	R	(S)	(S)	R	(S)	(S)	(S)	(S)
AUHPA368	O6	R1	F6	S	S	(S)	S	(S)	(S)	(S)	S
AUHPA369	O2/O5/O16/O18/O20	R2	F2	R	R	R	R	R	R	R	R
AUHPA371	O2/O5/O16/O18/O20	R2	F5	R	R	R	R	R	R	R	(S)
AUHPA372	O6	R1	F4	R	(S)	(S)	R	S	(S)	(S)	S
PA3596	O10/O19	R2	F5	R	(S)	(S)	(S)	R	(S)	S	(S)
PA3613	O6	R1	F5	S	R	(S)	R	(S)	S	S	R
PA3625	O2/O5/O16/O18/O20	R2	F2	S	R	S	R	R	S	S	R
PA3681	O4	R5	×	R	(S)	(S)	R	(S)	S	S	R

PA3712	O6	R1	×	R	S	(S)	S	(S)	R	R	S
PA3715	O3/O15	×	F2	S	S	(S)	(S)	(S)	S	S	(S)
PA3744	O11/O17	R5	×	(S)	(S)	(S)	R	(S)	S	S	S
PA3772	O3/O15	×	F2	S	S	(S)	(S)	(S)	R	R	(S)
AUHPA1	O6	R1	F4	(S)	(S)	(S)	R	S	R	R	(S)
AUHPA3	Others	R2	F11	S	(S)	(S)	R	(S)	S	S	(S)
AUHPA4	Others	R2	F5	R	R	(S)	R	R	(S)	(S)	(S)
AUHPA5	O6	×	F12	S	(S)	(S)	(S)	S	S	S	S
AUHPA6	O1	×	F2	S	S	S	S	(S)	(S)	R	S
AUHPA7	O6	×	F12	S	(S)	(S)	(S)	S	S	S	S
AUHPA8	O11/O17	R5	×	(S)	(S)	(S)	R	S	S	S	S
AUHPA9	O3/O15	×	F4	S	S	(S)	S	S	S	S	(S)
AUHPA10	O11/O17	R5	×	R	R	(S)	R	(S)	S	S	S
AUHPA11	O11/O17	R5	×	S	R	(S)	R	(S)	(S)	(S)	S
AUHPA12	O2/O5/O16/O18/O20	R2	F11	R	R	(S)	R	R	(S)	(S)	(S)
AUHPA13	O13/O14	R2	F7	(S)	R	R	(S)	S	R	R	R
AUHPA14	O11/O17	R5	×	R	R	R	S	(S)	S	S	S
AUHPA15	O7/O8	R2	×	R	R	(S)	R	R	S	S	S
AUHPA16	O6	R1	F6	R	S	(S)	(S)	S	(S)	(S)	S
AUHPA17	O6	R1	F6	(S)	R	R	(S)	(S)	R	R	S
AUHPA18	O4	R5	×	R	R	(S)	R	(S)	(S)	(S)	R
AUHPA19	O1	×	F2	S	(S)	(S)	R	(S)	R	R	S
AUHPA20	O1	NT	F2	R	R	(S)	R	(S)	(S)	(S)	S
AUHPA21	O6	R1	×	R	(S)	(S)	S	(S)	(S)	(S)	S
AUHPA22	O2/O5/O16/O18/O20	R2	F2	R	R	R	R	R	(S)	(S)	R
AUHPA23	O11/O17	R5	×	R	(S)	R	R	(S)	(S)	(S)	S
AUHPA24	O7/O8	R2	×	R	R	(S)	S	R	S	S	(S)
AUHPA25	O6	R1	×	R	S	(S)	S	S	S	S	(S)

AUHPA26	O1	×	F2	(S)	R	(S)	(S)	(S)	S	S	(S)
AUHPA27	O1	×	F2	(S)	S	(S)	R	R	(S)	(S)	S
AUHPA28	O11/O17	R5	×	R	R	(S)	R	(S)	S	S	S
AUHPA29	O2/O5/O16/O18/O20	R2	F11	R	R	(S)	R	R	(S)	(S)	(S)
AUHPA30	O1	NT	F12	R	(S)	(S)	R	(S)	R	R	S
AUHPA31	O2/O5/O16/O18/O20	R2	F12	R	R	(S)	R	R	R	R	S
AUHPA32	O6	×	F7	(S)	S	(S)	R	S	R	R	(S)
AUHPA33	O11/O17	R5	×	R	R	R	(S)	S	(S)	(S)	S
AUHPA34	O3/O15	×	F7	S	S	(S)	S	S	R	R	(S)
AUHPA35	O11/O17	R5	×	R	S	(S)	(S)	R	R	R	R
AUHPA36	O6	×	F1	S	S	(S)	R	S	S	S	S
AUHPA37	O2/O5/O16/O18/O20	R2	F2	R	R	(S)	R	R	R	R	R
AUHPA38	O13/O14	R1	×	R	S	R	R	R	R	R	R
AUHPA39	O1	NT	F2	(S)	R	S	(S)	S	S	S	(S)
AUHPA40	O2/O5/O16/O18/O20	R1	F2	(S)	S	(S)	(S)	(S)	S	S	(S)
AUHPA41	O6	R1	F9	R	S	R	R	S	(S)	(S)	R
AUHPA42	O2/O5/O16/O18/O20	R2	F11	R	R	R	R	R	R	(S)	R
AUHPA43	Others	R1	F5	R	R	(S)	R	R	(S)	(S)	(S)
AUHPA44	O6	R2	×	R	S	(S)	S	(S)	S	S	(S)
AUHPA45	O2/O5/O16/O18/O20	R1	F11	R	R	(S)	R	R	(S)	(S)	S
AUHPA46	O2/O5/O16/O18/O20	R1	F11	R	R	S	R	(S)	(S)	(S)	R
AUHPA47	O3/O15	×	NT	S	S	S	(S)	S	S	S	(S)
AUHPA48	O13/O14	×	×	R	R	(S)	(S)	S	R	R	R
AUHPA49	O4	R5	×	(S)	R	(S)	R	(S)	(S)	(S)	R
AUHPA50	O1	×	F2	S	S	(S)	R	R	(S)	(S)	S
AUHPA51	O1	×	F2	S	S	(S)	R	R	(S)	(S)	S
AUHPA52	O1	×	F2	(S)	S	(S)	R	R	(S)	(S)	S
AUHPA399	O11/O17	R5	×	(S)	R	(S)	R	S	S	S	S

S: sensitive (S): weak sensitive R: resistant

Table S4. Antimicrobial susceptibility testing of 50 isolates.

Strains	Antimicrobial susceptibility	Strains	Antimicrobial susceptibility
**AUHPA311**	Pseudomonas aeruginosa<br>Antimicrobial MIC (ug/ml)<br>S :Gentamicin(GM) <=2<br>S :Amikacin(AN) <=8<br>S :Ciprofloxacin(CIP) <=0.5<br>S :Levofloxacin(LVX) <=1<br>S :Ceftazidime(CAZ) 4<br>S :Cefepime(FEP) 4<br>S :Imipenem(IPM) 2<br>S :Meropenem(MEM) 1<br>S :Piperacillin/Tazobactam(TZP) 8/4<br>R :Trimethoprim/Sulfamethoxazole(SXT) >2/38<br>Gram's stain:Gram Negative Bacilli	**AUHPA345**	Pseudomonas aeruginosa<br>Antimicrobial MIC (ug/ml)<br>S :Gentamicin(GM) <=2<br>S :Amikacin(AN) <=8<br>S :Ciprofloxacin(CIP) <=0.5<br>S :Levofloxacin(LVX) <=1<br>S :Ceftazidime(CAZ) 4<br>S :Cefepime(FEP) 2<br>S :Imipenem(IPM) 2<br>S :Meropenem(MEM)<br>S :Piperacillin/Tazobactam(TZP) 8/4<br>R :Trimethoprim/Sulfamethoxazole(SXT) >2/38<br>Gram's stain:Gram Negative Bacilli
**AUHPA312**	Pseudomonas aeruginosa<br>Antimicrobial MIC (ug/ml)<br>S :Gentamicin(GM) <=2<br>S :Amikacin(AN) <=8<br>R :Ciprofloxacin(CIP) >2<br>R :Levofloxacin(LVX) >4<br>R :Ceftazidime(CAZ) >16<br>R :Cefepime(FEP) >16<br>R :Imipenem(IPM) >4<br>R :Meropenem(MEM) >4<br>R :Piperacillin/Tazobactam(TZP) >64/4<br>R :Trimethoprim/Sulfamethoxazole(SXT) 2/38<br>Gram's stain:Gram Negative Bacilli	**AUHPA346**	Pseudomonas aeruginosa<br>Antimicrobial MIC (ug/mL)<br>I :Levofloxacin(LVX) 2<br>S :Meropenem(MEM) 0.5<br>R :Gentamicin(GM) <=2<br>S :Ceftazidime(CAZ) 8<br>S :Imipenem(IPM) 2<br>S :Piperacillin/Tazobactam(TZP) 16/4<br>S :Ciprofloxacin(CIP) <=0.5<br>R :Trimethoprim/Sulfamethoxazole(SXT) >2/38<br>S :Cefepime(FEP) 8<br>Gram's stain:Gram Negative Bacilli
**AUHPA313**	Pseudomonas aeruginosa<br>Antimicrobial MIC (ug/ml)<br>S :Gentamicin(GM) <=2<br>S :Amikacin(AN) <=8<br>S :Ciprofloxacin(CIP) <=0.5<br>S :Levofloxacin(LVX) <=1<br>S :Ceftazidime(CAZ) 2<br>S :Cefepime(FEP) 2<br>S :Imipenem(IPM) 2<br>S :Meropenem(MEM) 1<br>S :Piperacillin/Tazobactam(TZP) <=4/4<br>R :Trimethoprim/Sulfamethoxazole(SXT) >2/38<br>Gram's stain:Gram Negative Bacilli	**AUHPA347**	Pseudomonas aeruginosa<br>Antimicrobial MIC (ug/mL)<br>S :Levofloxacin(LVX) <=1<br>S :Meropenem(MEM) <=0.25<br>R :Gentamicin(GM) <=2<br>S :Ceftazidime(CAZ) 2<br>S :Imipenem(IPM) 2<br>S :Piperacillin/Tazobactam(TZP) <=4/4<br>S :Ciprofloxacin(CIP) <=0.5<br>R :Trimethoprim/Sulfamethoxazole(SXT) 2/38<br>S :Cefepime(FEP) 2<br>Gram's stain:Gram Negative Bacilli
**AUHPA314**	Pseudomonas aeruginosa<br>Antimicrobial MIC (ug/ml)<br>S :Gentamicin(GM) <=2<br>S :Amikacin(AN) <=8<br>R :Ciprofloxacin(CIP) >2<br>R :Levofloxacin(LVX) >4<br>S :Ceftazidime(CAZ) 4<br>S :Cefepime(FEP) 4<br>R :Imipenem(IPM) >4<br>R :Meropenem(MEM) >4<br>S :Piperacillin/Tazobactam(TZP) 8/4<br>R :Trimethoprim/Sulfamethoxazole(SXT) 2/38<br>Gram's stain:Gram Negative Bacilli	**AUHPA348**	Pseudomonas aeruginosa<br>Antimicrobial MIC (ug/mL)<br>S :Levofloxacin(LVX) <=1<br>S :Meropenem(MEM) 0.5<br>R :Gentamicin(GM) 4<br>S :Ceftazidime(CAZ) 4<br>S :Imipenem(IPM) 2<br>S :Piperacillin/Tazobactam(TZP) <=4/4<br>S :Ciprofloxacin(CIP) <=0.5<br>R :Trimethoprim/Sulfamethoxazole(SXT) 2/38<br>S :Cefepime(FEP) 2<br>Gram's stain:Gram Negative Bacill

**AUHPA315**		Pseudomonas aeruginosa<br>Antimicrobial MIC (ug/ml)<br>S :Gentamicin(GM) <=2<br>S :Amikacin(AN) <=8<br>S :Ciprofloxacin(CIP) <=0.5<br>S :Levofloxacin(LVX) <=1<br>S :Ceftazidime(CAZ) 4<br>S :Cefepime(FEP) 4<br>S :Imipenem(IPM) 2<br>S :Meropenem(MEM) 0.5<br>S :Piperacillin/Tazobactam(TZP) 8/4<br>R :Trimethoprim/Sulfamethoxazole(SXT) >2/38<br>Gram's stain:Gram Negative Bacilli		**AUHPA349**		Pseudomonas aeruginosa<br>Antimicrobial MIC (ug/mL)<br>S :Levofloxacin(LVX) <=1<br>S :Meropenem(MEM) <=0.25<br>R :Gentamicin(GM) <=2<br>S :Ceftazidime(CAZ) 8<br>S :Imipenem(IPM) <=0.25<br>S :Piperacillin/Tazobactam(TZP) 16/4<br>S :Ciprofloxacin(CIP) <=0.5<br>R :Trimethoprim/Sulfamethoxazole(SXT) 1/19<br>S :Cefepime(FEP) 4<br>Gram's stain:Gram Negative Bacilli	
**AUHPA318**		Pseudomonas aeruginosa<br>Antimicrobial MIC (ug/ml)<br>S :Gentamicin(GM) <=2<br>S :Amikacin(AN) <=8<br>S :Ciprofloxacin(CIP) <=0.5<br>S :Levofloxacin(LVX) <=1<br>S :Ceftazidime(CAZ) 2<br>S :Cefepime(FEP) 2<br>S :Imipenem(IPM)<br>S :Meropenem(MEM) 2<br>S :Piperacillin/Tazobactam(TZP) <=4/4<br>R :Trimethoprim/Sulfamethoxazole(SXT) 2/38<br>Gram's stain:Gram Negative Bacilli		**AUHPA3410**		Pseudomonas aeruginosa<br>Antimicrobial MIC (ug/mL)<br>R :Levofloxacin(LVX) >4<br>S :Meropenem(MEM) 0.5<br>R :Gentamicin(GM) <=2<br>S :Ceftazidime(CAZ) 2<br>S :Imipenem(IPM) 1<br>S :Piperacillin/Tazobactam(TZP) <=4/4<br>R :Ciprofloxacin(CIP) >2<br>R :Trimethoprim/Sulfamethoxazole(SXT) 2/38<br>S :Cefepime(FEP) 8<br>Gram's stain:Gram Negative Bacilli	
**AUHPA319**		Pseudomonas aeruginosa<br>Antimicrobial MIC (ug/ml)<br>S :Gentamicin(GM) <=2<br>S :Amikacin(AN) <=8<br>S :Ciprofloxacin(CIP) <=0.5<br>S :Levofloxacin(LVX) <=1<br>S :Ceftazidime(CAZ) 4<br>S :Cefepime(FEP) 4<br>S :Imipenem(IPM) 2<br>S :Meropenem(MEM) 1<br>S :Piperacillin/Tazobactam(TZP) 8/4<br>R :Trimethoprim/Sulfamethoxazole(SXT) 2/38<br>Gram's stain:Gram Negative Bacilli		**AUHPA351**		Pseudomonas aeruginosa<br>Antimicrobial MIC (ug/mL)<br>S :Levofloxacin(LVX) <=1<br>S :Meropenem(MEM) 0.5<br>R :Gentamicin(GM) <=2<br>S :Ceftazidime(CAZ) 2<br>S :Imipenem(IPM) 2<br>S :Piperacillin/Tazobactam(TZP) <=4/4<br>S :Ciprofloxacin(CIP) <=0.5<br>R :Trimethoprim/Sulfamethoxazole(SXT) 2/38<br>S :Cefepime(FEP) 2<br>Gram's stain:Gram Negative Bacilli	
**AUHPA321**		Pseudomonas aeruginosa<br>Antimicrobial MIC (ug/ml)<br>S :Gentamicin(GM) <=2<br>S :Amikacin(AN) <=8<br>S :Ciprofloxacin(CIP) <=0.5<br>S :Levofloxacin(LVX) <=1<br>S :Ceftazidime(CAZ) 2<br>S :Cefepime(FEP) 4<br>S :Imipenem(IPM) 2<br>S :Meropenem(MEM) <=0.25<br>S :Piperacillin/Tazobactam(TZP) <=4/4<br>R :Trimethoprim/Sulfamethoxazole(SXT) 2/38<br>Gram's stain:Gram Negative Bacilli		**AUHPA352**		Pseudomonas aeruginosa<br>Antimicrobial MIC (ug/mL)<br>I :Levofloxacin(LVX) 2<br>S :Meropenem(MEM) 2<br>R :Gentamicin(GM) 4<br>S :Ceftazidime(CAZ) 4<br>S :Imipenem(IPM)<br>S :Piperacillin/Tazobactam(TZP) 8/4<br>I :Ciprofloxacin(CIP) 1<br>R :Trimethoprim/Sulfamethoxazole(SXT) >2/38<br>S :Cefepime(FEP) 4<br>Gram's stain:Gram Negative Bacilli	
**AUHPA322**		Pseudomonas aeruginosa<br>Antimicrobial MIC (ug/ml)<br>S :Gentamicin(GM) <=2<br>S :Amikacin(AN) <=8<br>S :Ciprofloxacin(CIP) <=0.5<br>S :Levofloxacin(LVX) <=1<br>S :Ceftazidime(CAZ) 4<br>S :Cefepime(FEP) 4<br>S :Imipenem(IPM) 2<br>S :Meropenem(MEM) <=0.25<br>S :Piperacillin/Tazobactam(TZP) 8/4<br>R :Trimethoprim/Sulfamethoxazole(SXT) >2/38<br>Gram's stain:Gram Negative Bacilli		**AUHPA354**		Pseudomonas aeruginosa<br>Antimicrobial MIC (ug/mL)<br>R :Levofloxacin(LVX) >4<br>R :Meropenem(MEM)<br>R :Gentamicin(GM) <=2<br>S :Ceftazidime(CAZ) 4<br>I :Imipenem(IPM) 4<br>S :Piperacillin/Tazobactam(TZP) 8/4<br>R :Ciprofloxacin(CIP) >2<br>R :Trimethoprim/Sulfamethoxazole(SXT) >2/38<br>S :Cefepime(FEP) 8<br>Gram's stain:Gram Negative Bacilli	

**AUHPA323**		Pseudomonas aeruginosa<br>Antimicrobial MIC (ug/ml)<br>S :Gentamicin(GM) <=2<br>S :Amikacin(AN) <=8<br>S :Ciprofloxacin(CIP) <=0.5<br>S :Levofloxacin(LVX) <=1<br>S :Ceftazidime(CAZ) 8<br>S :Cefepime(FEP) 4<br>S :Imipenem(IPM) 2<br>S :Meropenem(MEM) <=0.25<br>S :Piperacillin/Tazobactam(TZP) <=4/4<br>R :Trimethoprim/Sulfamethoxazole(SXT) >2/38<br>Gram's stain:Gram Negative Bacilli		**AUHPA355**		Pseudomonas aeruginosa<br>Antimicrobial MIC (ug/mL)<br>S :Levofloxacin(LVX) <=1<br>S :Meropenem(MEM) <=0.25<br>R :Gentamicin(GM) <=2<br>S :Ceftazidime(CAZ) 4<br>S :Imipenem(IPM)<br>S :Piperacillin/Tazobactam(TZP) <=4/4<br>S :Ciprofloxacin(CIP) <=0.5<br>R :Trimethoprim/Sulfamethoxazole(SXT) 2/38<br>S :Cefepime(FEP) 2<br>Gram's stain:Gram Negative Bacilli	
**AUHPA325**		Pseudomonas aeruginosa<br>Antimicrobial MIC (ug/ml)<br>R :Gentamicin(GM) >8<br>R :Amikacin(AN) >32<br>R :Ciprofloxacin(CIP) >2<br>R :Levofloxacin(LVX) >4<br>S :Ceftazidime(CAZ) 8<br>R :Cefepime(FEP) >16<br>I :Imipenem(IPM) 4<br>R :Meropenem(MEM) >4<br>I :Piperacillin/Tazobactam(TZP) 64/4<br>R :Trimethoprim/Sulfamethoxazole(SXT) >2/38<br>Gram's stain:Gram Negative Bacilli		**AUHPA356**		Pseudomonas aeruginosa<br>Antimicrobial MIC (ug/mL)<br>S :Levofloxacin(LVX) <=1<br>S :Meropenem(MEM) <=0.25<br>R :Gentamicin(GM) <=2<br>S :Ceftazidime(CAZ) 2<br>S :Imipenem(IPM) 1<br>S :Piperacillin/Tazobactam(TZP) <=4/4<br>S :Ciprofloxacin(CIP) <=0.5<br>R :Trimethoprim/Sulfamethoxazole(SXT) 2/38<br>S :Cefepime(FEP) 2<br>Gram's stain:Gram Negative Bacilli	
**AUHPA326**		Pseudomonas aeruginosa<br>Antimicrobial MIC (ug/ml)<br>I :Gentamicin(GM) 8<br>S :Amikacin(AN) <=8<br>S :Ciprofloxacin(CIP) <=0.5<br>R :Levofloxacin(LVX) 4<br>S :Ceftazidime(CAZ) 4<br>I :Cefepime(FEP) 16<br>S :Imipenem(IPM) 2<br>S :Meropenem(MEM) <=0.25<br>S :Piperacillin/Tazobactam(TZP) 8/4<br>R :Trimethoprim/Sulfamethoxazole(SXT) 2/38<br>Gram's stain:Gram Negative Bacilli		**AUHPA357**		Pseudomonas aeruginosa<br>Antimicrobial MIC (ug/mL)<br>S :Levofloxacin(LVX) <=1<br>S :Meropenem(MEM) 0.5<br>R :Gentamicin(GM) <=2<br>S :Ceftazidime(CAZ) 8<br>S :Imipenem(IPM) 1<br>S :Piperacillin/Tazobactam(TZP) 8/4<br>S :Ciprofloxacin(CIP) <=0.5<br>R :Trimethoprim/Sulfamethoxazole(SXT) 2/38<br>S :Cefepime(FEP) 4<br>Gram's stain:Gram Negative Bacilli	
**AUHPA331**		Pseudomonas aeruginosa<br>Antimicrobial MIC (ug/ml)<br>S :Gentamicin(GM) <=2<br>S :Amikacin(AN) <=8<br>S :Ciprofloxacin(CIP) <=0.5<br>S :Levofloxacin(LVX) <=1<br>S :Ceftazidime(CAZ) 2<br>S :Cefepime(FEP) 2<br>S :Imipenem(IPM) <=0.25<br>S :Meropenem(MEM) <=0.25<br>S :Piperacillin/Tazobactam(TZP) <=4/4<br>R :Trimethoprim/Sulfamethoxazole(SXT) >2/38<br>Gram's stain:Gram Negative Bacilli		**AUHPA359**		Pseudomonas aeruginosa<br>Antimicrobial MIC (ug/mL)<br>S :Levofloxacin(LVX) <=1<br>S :Meropenem(MEM) <=0.25<br>R :Gentamicin(GM) <=2<br>S :Ceftazidime(CAZ) 2<br>S :Imipenem(IPM) 2<br>S :Piperacillin/Tazobactam(TZP) 8/4<br>S :Ciprofloxacin(CIP) <=0.5<br>R :Trimethoprim/Sulfamethoxazole(SXT) >2/38<br>S :Cefepime(FEP) 4<br>Gram's stain:Gram Negative Bacilli	
**AUHPA332**		Pseudomonas aeruginosa<br>Antimicrobial MIC (ug/ml)<br>S :Gentamicin(GM) <=2<br>S :Amikacin(AN) <=8<br>S :Ciprofloxacin(CIP) <=0.5<br>S :Levofloxacin(LVX) <=1<br>S :Ceftazidime(CAZ) 2<br>S :Cefepime(FEP) 2<br>S :Imipenem(IPM) 2<br>S :Meropenem(MEM) 1<br>S :Piperacillin/Tazobactam(TZP) <=4/4<br>R :Trimethoprim/Sulfamethoxazole(SXT) 2/38<br>Gram's stain:Gram Negative Bacilli		**AUHPA3510**		Pseudomonas aeruginosa<br>Antimicrobial MIC (ug/mL)<br>S :Levofloxacin(LVX) <=1<br>S :Meropenem(MEM) 0.5<br>R :Gentamicin(GM) <=2<br>S :Ceftazidime(CAZ) 4<br>S :Imipenem(IPM) 2<br>S :Piperacillin/Tazobactam(TZP) <=4/4<br>S :Ciprofloxacin(CIP) <=0.5<br>R :Trimethoprim/Sulfamethoxazole(SXT) >2/38<br>S :Cefepime(FEP) 4<br>Gram's stain:Gram Negative Bacilli	

**AUHPA333**	Pseudomonas aeruginosa<br>Antimicrobial MIC (ug/ml)<br>S :Gentamicin(GM) <=2<br>S :Amikacin(AN) <=8<br>S :Ciprofloxacin(CIP) <=0.5<br>S :Levofloxacin(LVX) <=1<br>S :Ceftazidime(CAZ) 2<br>S :Cefepime(FEP) 2<br>S :Imipenem(IPM) 1<br>S :Meropenem(MEM) <=0.25<br>S :Piperacillin/Tazobactam(TZP) <=4/4<br>R :Trimethoprim/Sulfamethoxazole(SXT) >2/38<br>Gram's stain:Gram Negative Bacilli	**AUHPA361**	Pseudomonas aeruginosa<br>Antimicrobial MIC (ug/mL)<br>S :Levofloxacin(LVX) <=1<br>S :Meropenem(MEM) 1<br>R :Gentamicin(GM) <=2<br>S :Ceftazidime(CAZ) 2<br>S :Imipenem(IPM) 2<br>S :Piperacillin/Tazobactam(TZP) <=4/4<br>S :Ciprofloxacin(CIP) <=0.5<br>R :Trimethoprim/Sulfamethoxazole(SXT) 2/38<br>S :Cefepime(FEP) 4<br>Gram's stain:Gram Negative Bacilli
**AUHPA334**	Pseudomonas aeruginosa<br>Antimicrobial MIC (ug/ml)<br>S :Gentamicin(GM) <=2<br>S :Amikacin(AN) <=8<br>S :Ciprofloxacin(CIP) <=0.5<br>S :Levofloxacin(LVX) <=1<br>S :Ceftazidime(CAZ) 2<br>S :Cefepime(FEP) 2<br>S :Imipenem(IPM) 2<br>S :Meropenem(MEM) <=0.25<br>S :Piperacillin/Tazobactam(TZP) <=4/4<br>R :Trimethoprim/Sulfamethoxazole(SXT) 2/38<br>Gram's stain:Gram Negative Bacilli	**AUHPA362**	Pseudomonas aeruginosa<br>Antimicrobial MIC (ug/mL)<br>R :Levofloxacin(LVX) >4<br>S :Meropenem(MEM) 0.5<br>R :Gentamicin(GM) <=2<br>S :Ceftazidime(CAZ) 2<br>S :Imipenem(IPM)<br>S :Piperacillin/Tazobactam(TZP) <=4/4<br>R :Ciprofloxacin(CIP) >2<br>R :Trimethoprim/Sulfamethoxazole(SXT) >2/38<br>S :Cefepime(FEP) 2<br>Gram's stain:Gram Negative Bacilli
**AUHPA335**	Pseudomonas aeruginosa<br>Antimicrobial MIC (ug/ml)<br>S :Gentamicin(GM) <=2<br>S :Amikacin(AN) <=8<br>S :Ciprofloxacin(CIP) <=0.5<br>S :Levofloxacin(LVX) <=1<br>S :Ceftazidime(CAZ) 4<br>S :Cefepime(FEP) 4<br>R :Imipenem(IPM) >4<br>R :Meropenem(MEM) >4<br>S :Piperacillin/Tazobactam(TZP) 8/4<br>R :Trimethoprim/Sulfamethoxazole(SXT) >2/38<br>Gram's stain:Gram Negative Bacilli	**AUHPA363**	Pseudomonas aeruginosa<br>Antimicrobial MIC (ug/mL)<br>S :Levofloxacin(LVX) <=1<br>S :Meropenem(MEM) 0.5<br>R :Gentamicin(GM) <=2<br>S :Ceftazidime(CAZ) 4<br>S :Imipenem(IPM) 2<br>S :Piperacillin/Tazobactam(TZP) <=4/4<br>S :Ciprofloxacin(CIP) <=0.5<br>R :Trimethoprim/Sulfamethoxazole(SXT) 2/38<br>S :Cefepime(FEP) 4<br>Gram's stain:Gram Negative Bacilli
**AUHPA336**	Pseudomonas aeruginosa<br>Antimicrobial MIC (ug/ml)<br>S :Gentamicin(GM) <=2<br>S :Amikacin(AN) <=8<br>S :Ciprofloxacin(CIP) <=0.5<br>S :Levofloxacin(LVX) <=1<br>S :Ceftazidime(CAZ) 2<br>S :Cefepime(FEP) <=1<br>S :Imipenem(IPM)<br>S :Meropenem(MEM) <=0.25<br>S :Piperacillin/Tazobactam(TZP) <=4/4<br>R :Trimethoprim/Sulfamethoxazole(SXT) >2/38<br>Gram's stain:Gram Negative Bacilli	**AUHPA365**	Pseudomonas aeruginosa<br>Antimicrobial MIC (ug/mL)<br>S :Levofloxacin(LVX) <=1<br>S :Meropenem(MEM) 1<br>R :Gentamicin(GM) <=2<br>S :Ceftazidime(CAZ) 2<br>S :Imipenem(IPM)<br>S :Piperacillin/Tazobactam(TZP) <=4/4<br>S :Ciprofloxacin(CIP) <=0.5<br>R :Trimethoprim/Sulfamethoxazole(SXT) 2/38<br>S :Cefepime(FEP) 2<br>Gram's stain:Gram Negative Bacilli
**AUHPA337**	Pseudomonas aeruginosa<br>Antimicrobial MIC (ug/ml)<br>S :Gentamicin(GM)<br>S :Amikacin(AN)<br>S :Ciprofloxacin(CIP)<br>S :Levofloxacin(LVX)<br>S :Ceftazidime(CAZ)<br>S :Cefepime(FEP)<br>S :Imipenem(IPM)<br>S :Meropenem(MEM)<br>S :Piperacillin/Tazobactam(TZP)<br>R :Trimethoprim/Sulfamethoxazole(SXT)<br>Gram's stain:Gram Negative Bacilli	**AUHPA366**	Pseudomonas aeruginosa<br>Antimicrobial MIC (ug/mL)<br>S :Levofloxacin(LVX) <=1<br>S :Meropenem(MEM) 0.5<br>R :Gentamicin(GM) <=2<br>R :Ceftazidime(CAZ) >16<br>S :Imipenem(IPM) 2<br>R :Piperacillin/Tazobactam(TZP) >64/4<br>S :Ciprofloxacin(CIP) <=0.5<br>R :Trimethoprim/Sulfamethoxazole(SXT) 2/38<br>R :Cefepime(FEP) >16<br>Gram's stain:Gram Negative Bacilli

**AUHPA339**	Pseudomonas aeruginosa<br>Antimicrobial MIC (ug/ml)<br>S :Gentamicin(GM) <=2<br>S :Amikacin(AN) <=8<br>S :Ciprofloxacin(CIP) <=0.5<br>S :Levofloxacin(LVX) <=1<br>S :Ceftazidime(CAZ) 4<br>S :Cefepime(FEP) 4<br>S :Imipenem(IPM) 2<br>S :Meropenem(MEM) 0.5<br>S :Piperacillin/Tazobactam(TZP) <=4/4<br>R :Trimethoprim/Sulfamethoxazole(SXT) >2/38<br>Gram's stain:Gram Negative Bacilli	**AUHPA367**	Pseudomonas aeruginosa<br>Antimicrobial MIC (ug/mL)<br>S :Levofloxacin(LVX) <=1<br>S :Meropenem(MEM) <=0.25<br>R :Gentamicin(GM) <=2<br>S :Ceftazidime(CAZ) 2<br>S :Imipenem(IPM) 2<br>S :Piperacillin/Tazobactam(TZP) <=4/4<br>S :Ciprofloxacin(CIP) <=0.5<br>R :Trimethoprim/Sulfamethoxazole(SXT) >2/38<br>S :Cefepime(FEP) 4<br>Gram's stain:Gram Negative Bacilli
**AUHPA3310**	Pseudomonas aeruginosa<br>Antimicrobial MIC (ug/ml)<br>S :Gentamicin(GM) <=2<br>S :Amikacin(AN) <=8<br>S :Ciprofloxacin(CIP) <=0.5<br>S :Levofloxacin(LVX) <=1<br>S :Ceftazidime(CAZ) 4<br>S :Cefepime(FEP) 4<br>S :Imipenem(IPM) 2<br>S :Meropenem(MEM) 0.5<br>S :Piperacillin/Tazobactam(TZP) 8/4<br>R :Trimethoprim/Sulfamethoxazole(SXT) 2/38<br>Gram's stain:Gram Negative Bacilli	**AUHPA368**	Pseudomonas aeruginosa<br>Antimicrobial MIC (ug/mL)<br>R :Levofloxacin(LVX) >4<br>I :Meropenem(MEM) 4<br>R :Gentamicin(GM) <=2<br>R :Ceftazidime(CAZ) >16<br>R :Imipenem(IPM) >4<br>R :Piperacillin/Tazobactam(TZP) >64/4<br>R :Ciprofloxacin(CIP) >2<br>R :Trimethoprim/Sulfamethoxazole(SXT) >2/38<br>I :Cefepime(FEP) 16<br>Gram's stain:Gram Negative Bacilli
**AUHPA341**	Pseudomonas aeruginosa<br>Antimicrobial MIC (ug/ml)<br>S :Gentamicin(GM) <=2<br>S :Amikacin(AN) <=8<br>S :Ciprofloxacin(CIP) <=0.5<br>S :Levofloxacin(LVX) <=1<br>S :Ceftazidime(CAZ) 4<br>S :Cefepime(FEP) 4<br>S :Imipenem(IPM) 2<br>S :Meropenem(MEM) <=0.25<br>S :Piperacillin/Tazobactam(TZP) <=4/4<br>R :Trimethoprim/Sulfamethoxazole(SXT) >2/38<br>Gram's stain:Gram Negative Bacilli	**AUHPA369**	Pseudomonas aeruginosa<br>Antimicrobial MIC (ug/mL)<br>S :Levofloxacin(LVX) <=1<br>S :Meropenem(MEM) 1<br>R :Gentamicin(GM) <=2<br>S :Ceftazidime(CAZ) 2<br>S :Imipenem(IPM) 2<br>S :Piperacillin/Tazobactam(TZP) <=4/4<br>S :Ciprofloxacin(CIP) <=0.5<br>R :Trimethoprim/Sulfamethoxazole(SXT) 2/38<br>S :Cefepime(FEP) 4<br>Gram's stain:Gram Negative Bacilli
**AUHPA342**	Pseudomonas aeruginosa<br>Antimicrobial MIC (ug/ml)<br>S :Gentamicin(GM) <=2<br>S :Amikacin(AN) <=8<br>S :Ciprofloxacin(CIP) <=0.5<br>S :Levofloxacin(LVX) <=1<br>S :Ceftazidime(CAZ) 4<br>S :Cefepime(FEP) 4<br>S :Imipenem(IPM) 2<br>S :Meropenem(MEM) <=0.25<br>S :Piperacillin/Tazobactam(TZP) <=4/4<br>R :Trimethoprim/Sulfamethoxazole(SXT) >2/38<br>Gram's stain:Gram Negative Bacilli	**AUHPA371**	Pseudomonas aeruginosa<br>Antimicrobial MIC (ug/mL)<br>S :Levofloxacin(LVX) <=1<br>S :Meropenem(MEM) 0.5<br>R :Gentamicin(GM) <=2<br>S :Ceftazidime(CAZ) 2<br>S :Imipenem(IPM) 2<br>S :Piperacillin/Tazobactam(TZP) <=4/4<br>S :Ciprofloxacin(CIP) <=0.5<br>R :Trimethoprim/Sulfamethoxazole(SXT) >2/38<br>S :Cefepime(FEP) 2<br>Gram's stain:Gram Negative Bacilli
**AUHPA343**	Pseudomonas aeruginosa<br>Antimicrobial MIC (ug/ml)<br>S :Gentamicin(GM) <=2<br>S :Amikacin(AN) <=8<br>S :Ciprofloxacin(CIP) <=0.5<br>S :Levofloxacin(LVX) <=1<br>S :Ceftazidime(CAZ) 4<br>S :Cefepime(FEP) 4<br>S :Imipenem(IPM) 1<br>S :Meropenem(MEM) 0.5<br>S :Piperacillin/Tazobactam(TZP) 8/4<br>R :Trimethoprim/Sulfamethoxazole(SXT) >2/38<br>Gram's stain:Gram Negative Bacilli	**AUHPA372**	Pseudomonas aeruginosa<br>Antimicrobial MIC (ug/mL)<br>R :Levofloxacin(LVX) >4<br>S :Meropenem(MEM) <=0.25<br>R :Gentamicin(GM) <=2<br>S :Ceftazidime(CAZ) 2<br>S :Imipenem(IPM)<br>S :Piperacillin/Tazobactam(TZP) <=4/4<br>R :Ciprofloxacin(CIP) >2<br>R :Trimethoprim/Sulfamethoxazole(SXT) >2/38<br>S :Cefepime(FEP) 4<br>Gram's stain:Gram Negative Bacilli

**AUHPA344**	Pseudomonas aeruginosa<br>Antimicrobial MIC (ug/ml)<br>S :Gentamicin(GM) <=2<br>S :Amikacin(AN) <=8<br>S :Ciprofloxacin(CIP) <=0.5<br>S :Levofloxacin(LVX) <=1<br>S :Ceftazidime(CAZ) 2<br>S :Cefepime(FEP) 4<br>S :Imipenem(IPM) 2<br>S :Meropenem(MEM) 0.5<br>S :Piperacillin/Tazobactam(TZP) <=4/4<br>R :Trimethoprim/Sulfamethoxazole(SXT) >2/38<br>Gram's stain:Gram Negative Bacilli	**AUHPA399**	Pseudomonas aeruginosa<br>Antimicrobial MIC (ug/mL)<br>R :Levofloxacin(LVX) 4<br>R :Meropenem(MEM) >4<br>R :Gentamicin(GM) <=2<br>S :Ceftazidime(CAZ) 8<br>R :Imipenem(IPM) >4<br>I :Piperacillin/Tazobactam(TZP) 32/4<br>S :Ciprofloxacin(CIP) <=0.5<br>R :Trimethoprim/Sulfamethoxazole(SXT) >2/38<br>I :Cefepime(FEP) 16<br>Gram's stain:Gram Negative Bacilli

Table S5. Minimum inhibitory concentrations (MICs) of pyocin subtypes against AUHPA312, AUHPA325 and AUHPA399.

	MIC (mg/mL)		
Pyocins	Strains		
	AUHPA312 (O6)	AUHPA325 (O11/O17)	AUHPA399 (O11/O17)
R1	-	~0.1	0.3
R2	~0.008	-	-
R5	~0.004	-	0.3
F1	~0.0005	~0.3	-
F2	~0.004	~0.0005	0.0002
F4	~0.00002	~0.004	0.00003
F7	~0.00002	~0.004	0.0002
F12	~0.06	~0.00003	0.00003
R1/F1	-	~0.25	-
R1/F2	-	~0.00025	0.0001
R1/F4	-	~0.002	0.000015
R1/F7	-	~0.002	0.0002
R1/F12	-	~0.000015	0.0001
R2/F1	~0.0001	-	-
R2/F2	~0.00025	-	-
R2/F4	~0.00001	-	-
R2/F7	~0.00001	-	-
R2/F12	~0.002	-	-
R5/F1	~0.0001	-	-
R5/F2	~0.00025	-	0.001
R5/F4	~0.00001	-	0.0002
R5/F7	~0.00001	-	0.00006
R5/F12	~0.002	-	0.0002

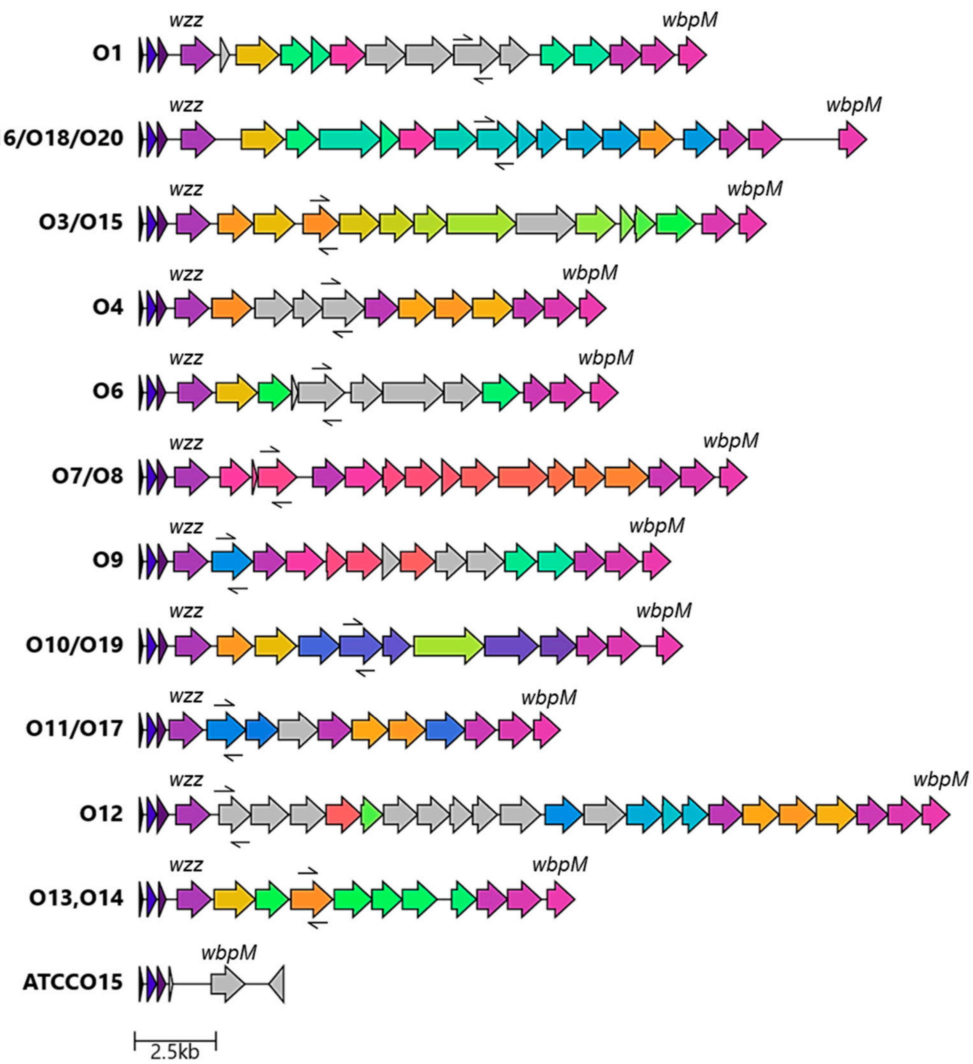

Figure S1. A schematic diagram of the 11 groups of gene clusters involved in the OSA biosynthesis. Proteins with similar encoded sequences are marked with the same color. The black arrows indicate the primer pairs for serotyping the *P. aeruginosa* isolates using PCR and DNA sequencing. This diagram was adapted from Raymond et al. [1] and generated using CAGECAT [2]. The sequences of the primer pairs used in the study are summarized in Supplementary Table S1.

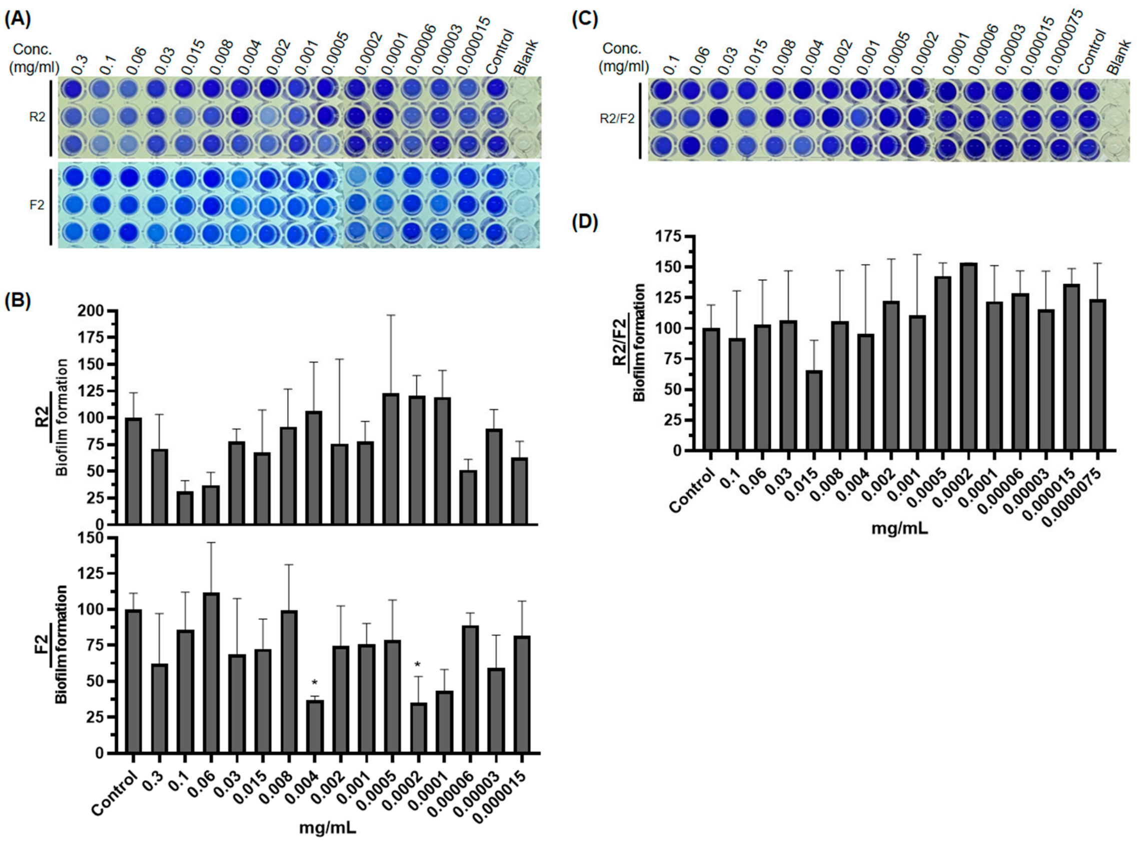

Figure S2. Biofilm development of AUHPA312 after pyocin treatments. (A, C) Crystal violet assays showing biofilm formation of AUHPA399 after treatments of (A) individual pyocins, R2 and F2, and (C) R2/F2 cocktail. The highest concentrations of added pyocin are indicated, followed by a 2-fold serial dilution. Control: no pyocin added. (B, D) Normalized level of biofilm formation of AUHPA312 in 2-fold serially diluted (B) individual pyocins R2 and F2, and (D) R2/F2 cocktail. All the OD_{570} values in each well are normalized against the control as 100%. The statistical significance between the OD_{570} values for the control and pyocin-treated cells was analyzed by one-way ANOVA using GraphPad Prism. Data represent the mean ± SD with $n = 3$. *$p < 0.05$, **$p < 0.01$.

References

1. Raymond, C. K., Sims, E. H., Kas, A., Spencer, D. H., Kutyavin, T. V., Ivey, R. G., Zhou, Y., Kaul, R., Clendenning, J. B. & Olson, M. V. (2002). Genetic variation at the O-antigen biosynthetic locus in Pseudomonas aeruginosa. J Bacteriol. **184**, 3614-3622. https://doi.org/10.1128/jb.184.13.3614-3622.2002.
2. van den Belt, M., Gilchrist, C., Booth, T. J., Chooi, Y. H., Medema, M. H. & Alanjary, M. (2023). CAGECAT: The CompArative GEne Cluster Analysis Toolbox for rapid search and visualisation of homologous gene clusters. BMC Bioinformatics. **24**, 181. https://doi.org/10.1186/s12859-023-05311-2.
